# An atlas of exposome–phenome associations in health and disease risk

**DOI:** 10.1038/s41591-026-04266-0

**Published:** 2026-03-18

**Authors:** Chirag J. Patel, John P. A. Ioannidis, Arjun K. Manrai

**Affiliations:** 1 Department of Biomedical Informatics, Harvard Medical School, Boston, MA USA; 2 Department of Medicine, Department of Epidemiology and Population Health, Department of Biomedical Data Science, Stanford University, Stanford, CA USA; 3 Meta-Research Innovation Center at Stanford, Stanford University, Stanford, CA USA

**Keywords:** Predictive markers, Risk factors, Epidemiology

## Abstract

Nongenetic exposures comprising the ‘exposome’, including diet, lifestyle, infections and pollutants, shape many clinical phenotypes yet the evidence remains fragmented. Here we conducted an exposome-wide association study incorporating 619 exposure indicators and 305 quantitative phenotypes across ten independent waves of the US Centers for Disease Control and Prevention National Health and Nutrition Examination Survey. Replicable and stable signals were most concentrated in cardiometabolic and anthropometric phenotypes, linking objective nutrient biomarkers and lipophilic pollutants with body mass index, glycated hemoglobin and lipid profiles. Triglycerides, an important marker for cardiovascular risk, emerged as the phenotype most strongly associated with multidomain exposures, notably trans fatty acids, persistent pollutants and vitamin E isoforms. In pulmonary traits, tobacco-specific and carcinogen biomarkers were more prominently associated with reduced lung function than short-lived nicotine metabolites, refining exposomic links to forced expiratory volume in 1 s. Whereas individual exposures showed modest effects, aggregate ‘poly-exposomic’ models explained phenotypic variation comparable to genome-wide polygenic scores. Exposome globes further reveal an interconnected architecture where exposures rarely act in isolation, complicating causal attribution while providing a more holistic view of environmental risk. Our findings highlight which exposures are most likely to add value to disease risk assessment, population surveillance as well as further exposure prioritization and next-generation longitudinal exposomics.

## Abstract (web summary)

An exposome-wide association study of 619 exposures and 305 quantitative phenotypes shows that blood lipids, persistent pollutants and vitamin E are key contributors to phenotypic health traits.

## Main

Clinically relevant phenotypes are influenced by both genetics and environmental exposures^1–3^. Despite this, the structural relationship between the exposome—defined as the totality of environmental exposures in broad physical, chemical and psychosocial domains^4,5^—and human health remains obscure, characterized by a lack of systematic mapping across its broad domains. Until now, interrogating exposome–phenome relationships has been limited to studies that target a few candidate exposures and phenotypes. These candidate studies are presented selectively in millions of papers on claimed associations yielding fragmented and often biased snapshots of the exposome–phenotype maze^6^. While candidate approaches have been successful in identifying factors with large effects, such as smoking^7^, these millions of studies so far have not yielded robust associations^8^; moreover, many reported results might be false positives^9^. For example, disciplines such as nutritional epidemiology have yielded numerous associations regarding single dietary factors and patterns in disease outcomes have been nonrobust^10,11^. Analogous debates have been made in fields studying other domains of the exposome, such as environmental epidemiology^12,13^. Previous criteria to gauge causality, such as those developed by Bradford and Hill^7^, may not be readily applicable in new exposome epidemiology scenarios, for example, if most of the true associations to be discovered have small effect sizes and not readily discernible biological plausibility^14^, analogy, coherence and specificity and there is no possibility to validate in experimental studies. Consequently, the opportunity to integrate environmental data into precision medicine remains underrealized.

Precision medicine approaches, however, are dominated by genetic factors. Which exposures, if measured, would meaningfully improve risk stratification or refine prognosis and how large are those effects relative to demographics and genetics? Many phenotypes routinely used for care, diagnosis, staging and risk prediction, such as lipids (for example, triglycerides), hemoglobin A1C% (A1C%) and fasting glucose, estimated glomerular rate (eGFR)/creatinine, inflammatory markers (for example, C-reactive protein (CRP)), and spirometry (forced expiratory respiratory volume in 1 s (FEV₁)), may be partially driven by modifiable exposures. Prioritizing clinical phenotypes by the magnitude and replicability of associations, contextualizing connections between smoking and nutrient biomarkers and quantifying variance explained to gauge utility for risk equations are needed for evaluating the role of the exposome in precision medicine.

Here, we hypothesize that the exposome exhibits a replicable associational architecture where aggregate factors explain clinically relevant phenotypic variance and disease risk. To evaluate this, we systematically quantify these relationships, executing an ‘exposome-wide association study’, establishing the data-driven foundation required to integrate the exposome into precision medicine^15^.

## Results

**Fig. 1:**
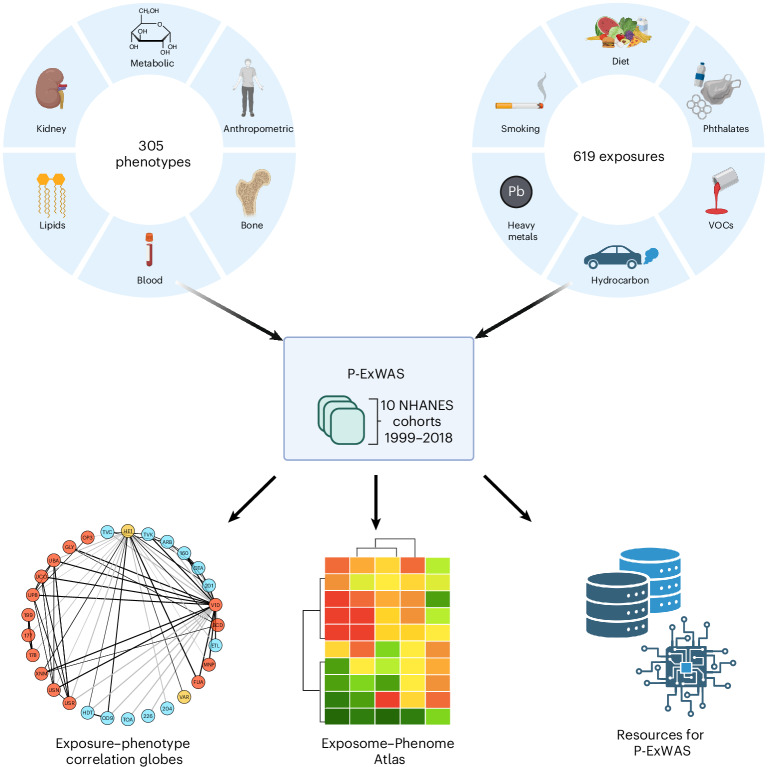
Schematic of conducting systematic P-ExWAS. Top left: Samples of the phenotypic domain comprising 305 phenotypes. Top right: Samples of the exposomic domain comprising 619 exposures. These data are harmonized across eight cohort samples of the NHANES 1999–2018. Bottom: Resources to describe the architecture of phenome–exposome associations, including exposome globes, the Exposome–Phenome Atlas and digital resources for conducting P-ExWAS (database and software). Figure created in BioRender; Patel, C. https://biorender.com/d4u5v1p (2026).

**Extended Data Fig. 1:**
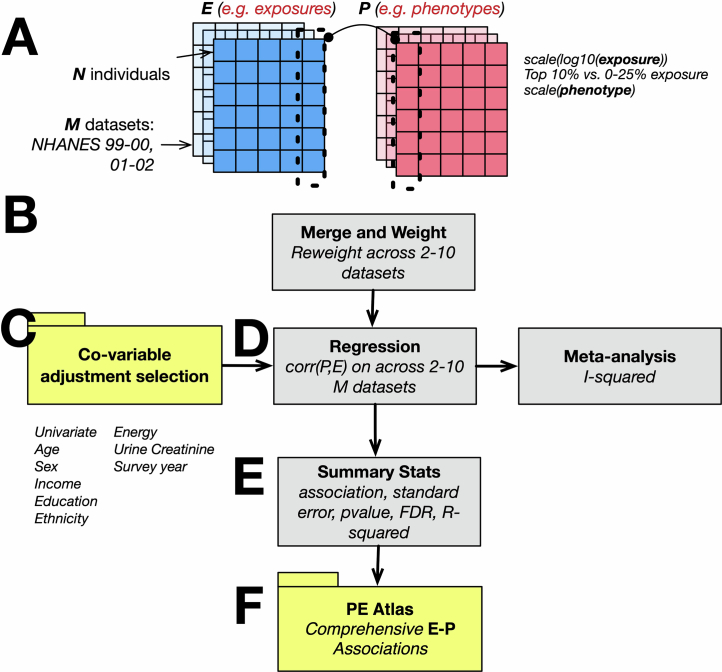
Methods flow for estimating exposomic architecture. **A**.) Merging datasets to combine exposures and phenotypes. Phenotypes and exposures may be transformed (right panel). **B**.) Reweighting the data to reflect appropriate sample weighting across 2-10 survey samplings. **C**.) Iterating through co-variable adjustments. A different combination of co-variables are chosen from the left hand side. Energy and urine creatinine are added for dietary and urinary biomarker associations, respectively. Survey year is added to all associations. **D**.) Survey weighted linear regression across 2-10 survey samplings. I^2^ is calculated to estimate heterogeneity. **E**.) Summary statistics are collected, including association size, p-values, and R^2^ for each phenotype-exposure association. **F**.) A web app atlas is available for download and browsing the atlas.

In brief, we developed an analytic pipeline (Fig. 1 and Extended Data Fig. 1) to analyze data from participants of the US National Health and Nutrition Examination Survey (NHANES)^16^ in ten serial cross-sectional surveys that were sampled in years 1999–2000, 2001–2002, 2003–2004, 2005–2006, 2007–2008, 2009–2010, 2011–2012, 2013–2014, 2015–2016 and 2017–2018. We cataloged a total of 374 real-valued continuous phenotypes and 810 biomarkers or self-report questionnaire responses that measure pollutant, dietary, infectious or smoking-related exposures across all ten surveys. Supplementary Fig. 1 shows the distribution of demographic characteristics (sex, age, ethnicity, education and income) for each association. The median age was 40 (interquartile range (IQR), 34 to 42) years and the median income-to-poverty ratio was 2.9 (IQR, 2.8 to 2.9). All associations are presented in Supplementary Table 1. The exposure and phenotype catalogs are presented in Supplementary Tables 2 and 3. Examples of exposures and phenotypes are presented in Supplementary Tables 4 and 5.

### Exposure-wide associations across the phenome

We conducted a ‘phenotype-by-exposome-wide association study’ (P-ExWAS), whereby each exposure is related with each phenotype^15^. We used survey-weighted regression to associate phenotypes with all exposures under nine different modeling scenarios that adjust for demographic and social attributes: the (1) main reported model, which consists of age, age^2^, sex, income (household income index divided by the poverty level), ethnicity (five groups), education (three groups: above high school, high school equivalent and below high school) and survey year (for example, 1999–2000, 2001–2002, 2003–2004, 2005–2006, 2007–2008, 2009–2010, 2010–2011, 2012–2013, 2014–2015 and 2016–2017 as a categorical variable); (2) base model, with no adjustments; (3) sex and survey year; (4) age, age^2^ and survey year; (5) sex, age, age^2^ and survey year; (6) ethnicity and survey year; (7) income, education and survey year (8) age, age^2^, sex, ethnicity and survey year; and (9) age, age^2^, sex, income, education and survey year.

**Fig. 2:**
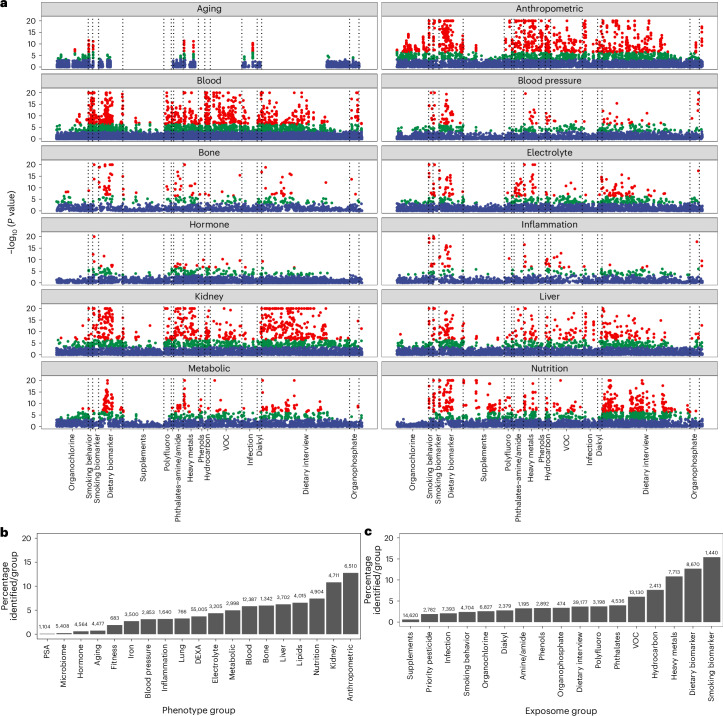
Associational architecture of the exposome on the phenome. **a**, The two-sided log_10_(*P* value) versus the exposure type. *P* values are not shown corrected for multiple hypotheses. Red, associations below Bonferroni (4 × 10^−7^); green, associations below the FDR (5 × 10^−4^) but greater than Bonferroni; blue, associations greater than the FDR. **b**, Number of significant phenotype–exposure associations per phenotype category (total *E* associations shown in text above bar). **c**, Number of significant phenotype–exposure associations per exposome category (total phenotype associations shown in text). Source data

**Fig. 3:**
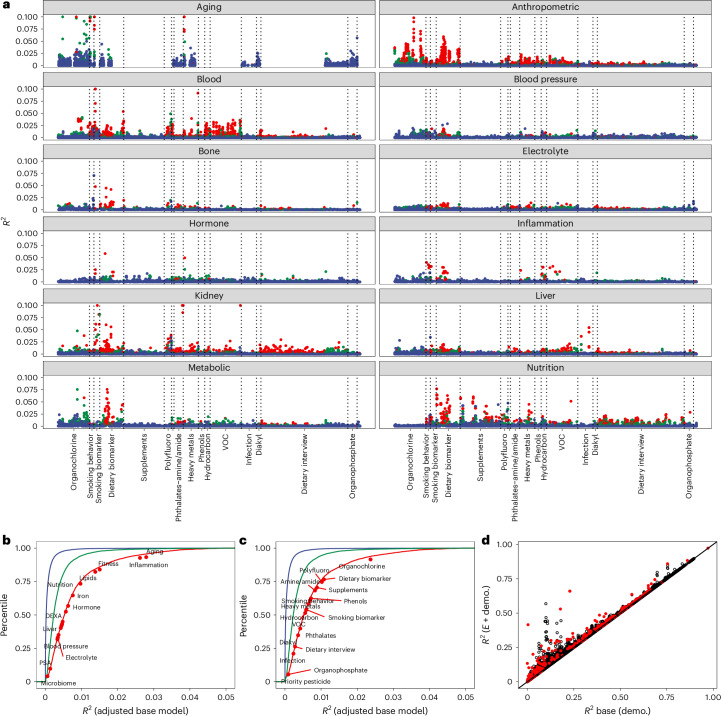
Variance explained by the exposome across phenotype groups. **a**, *R*^2^ for exposure across exposure categories and phenome categories. **b**, Cumulative distribution of *R*^2^. The median *R*^2^ for each phenotypic category is annotated. **c**, Cumulative distribution of *R*^2^ across 119k phenotype–exposure associations. The median *R*^2^ for each exposome category is annotated. Colors for **a** to **c**: red, associations below Bonferroni (4 × 10^−7^); green, associations below the FDR (5 × 10^−4^) but greater than Bonferroni; blue, associations greater than the FDR. **d**, *R*^2^ attributable to the exposure versus only to demographics (age, age^2^, ethnicity, income, education and sex). Red, *R*^2^ attributable to multiple simultaneous exposures (up to ten). Demo., demographics (age, age^2^, ethnicity, income, education and sex). Source data

**Fig. 4:**
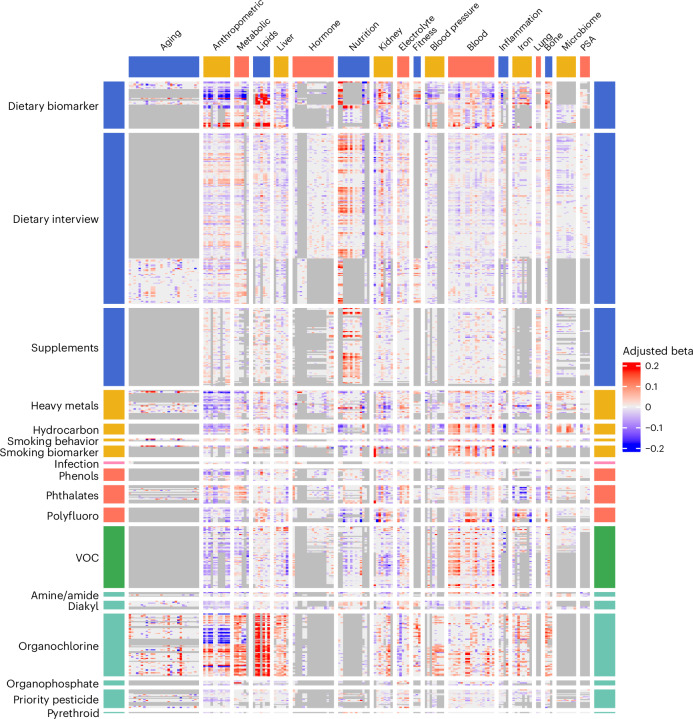
Phenome–Exposome Atlas. A total of 305 phenotypes across 18 categories are depicted in the columns and 625 exposures across 18 categories are depicted in the rows. Each entry in the matrix is the linear association (‘adjusted beta’) between exposure and phenotype. Gray shading denotes associations that could not be estimated owing to pairwise missingness or a total sample size lower than 500. Source data

We scaled all continuous exposures and phenotypes by their standard deviation (s.d.; Methods) and ran regression models to obtain standardized β-coefficients, *P* values and *R*^2^ (Figs. 2–4). Categorical exposures were compared with predefined reference groups. Statistical significance was defined by a Bonferroni threshold (*α* ≈ 4 × 10^−7^) and the Benjamini–Yekutieli false discovery rate (FDR).

The number of associations across all phenotype–exposure associations that passed the Bonferroni threshold was 5,674 (5% of 123,774) (Fig. 2a, blue shaded points, and Supplementary Fig. 2). The *P* value corresponding to an FDR of 5% was 5.1 × 10^−4^ (Fig. 2a, blue and red shaded points). The total number of associations that exceeded the FDR of 5% was 15,386 (12%).

The total number of tests conducted per phenotype (*n* = 305) was 16–654 (median of 397). The average percentage of associations (per phenotype) that were Bonferroni significant was 5% (range of 0.25–20%). The most associations were found for serum bilirubin, waist circumference and body mass index (BMI; 20% of tests for these phenotypes were significant for over 640 total tests).

We observed a large range of identified associations by exposome or phenome category (Fig. 2b,c). For example, the anthropometric phenome category saw the highest number of associations (13% of phenotypes in this category had a Bonferroni significant association; Fig. 2b and Supplementary Fig. 2). Of the exposome variables, smoking and dietary/nutrient biomarkers were implicated in the most phenotype–exposure associations: ~15% and 13%, respectively (Fig. 2c and Supplementary Fig. 2).

### Phenotype–exposure associations replicate across cohorts

For the reporting of main results, we combined cohort samplings across survey waves to maximize power. We can also examine associations within each of the survey samples to estimate the approximate ‘replication rate’. We estimated the rate of ‘replication’ or the number of times an association appeared to be nominally significant at a *P* value threshold of 0.05 in greater than one survey sampling, which we call a ‘replication rate’. Of the 5,674 associations that were Bonferroni significant, the replication rate was 41% (*n* = 2,321). By contrast, for those that did not achieve an FDR nor Bonferroni significance, the replication rate was 0.8% (*n* = 867).

**Extended Data Fig. 2:**
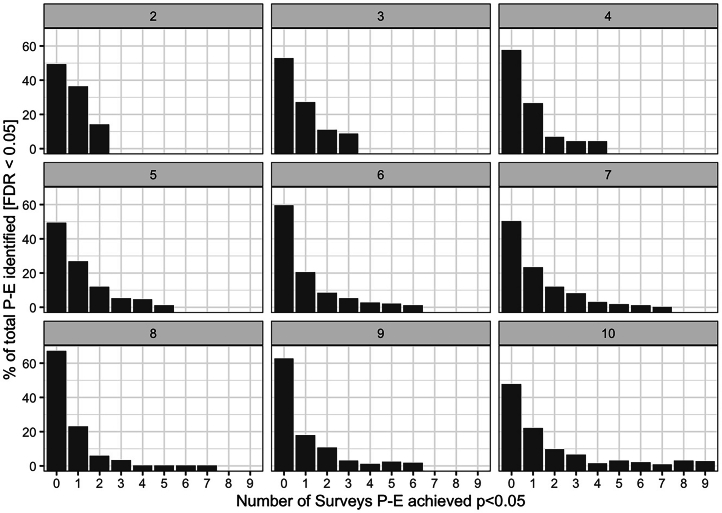
Exposure-phenotype associations across model specifications (adjustment choice). **A**.) We chose Bonferroni significant associations from a fully specified model with Age, Sex, Ethnicity, Income, Education, and Survey Year as covariates and compared those to ‘univariate’ (for example, only consider the exposure), age+exposure, sex + exposure, age+sex+exposure, age+sex+ethnicity, income + education + exposure, age + sex + income + education + exposure, age + sex + ethnicity. Distributions show the difference between phenotype-exposure associations from a fully specified model to those with other configurations. **B**.) Association for exposures associated with Body Mass Index under different scenarios. Cadmium shows both positive and negative association under different covariate scenarios. Data are presented as the association (solid dot) and the horizontal bars depict the 95% confidence interval, which is 1.96 times the standard error of the association. Source data

Across the atlas of associations, we found that an association (at a *P* value threshold of 0.05) occurred in two surveys 5% of the time. By contrast, if a phenotype–exposure association achieved an FDR significance across all surveys (Fig. 2), *P* value significance was achieved in greater than two surveys at least 20% of the time (Extended Data Fig. 2). However, phenotype–exposure replicated rates vary depending on the number of surveys available for a phenotype–exposure association. Specifically, for FDR-significant phenotype–exposure associations assessed in only two surveys were found in both surveys 39% of the time at a *P* value less than 0.05 (Extended Data Fig. 2). For associations that were at least FDR significant, the median *I*^2^ was 0, 5, 0, 26, 6, 14, 14, 20 and 18% for associations in 2, 3, 4, 5, 6, 7, 8, 9 and 10 surveys. We also assessed the percentage that were nominally significant in multiple survey waves. For the 1,211 associations estimated in ten survey waves (for example, they were tested in each of the ten surveys), there were 76%, 11%, 4%, 2.5%, 1%, 1%, 1%, 0.5%, 1% and 1% of associations that were nominally significant in exactly 0, 1, 2, 3, 4, 5, 6, 7, 8, 9 or 10 surveys. In other words, the replication rate among 1,211 associations estimated in ten different cohort samples was 13% (*n* = 161/1,211).

See Supplementary Fig. 3 for heterogeneity of association across survey samples.

### Variance explained of the exposome

We estimated the variance explained (*R*^2^) attributable to the exposure variable (after subtracting the potential role of demographic attributes; Fig. 3b–d and Methods). Demographic factors, including age, age^2^, ethnicity, income, education and sex explained a large range of overall phenotypic variation, from ~0% to 80% (Fig. 3d, *x* axis). In comparison, single exposures added a median of 0.14% (Fig. 3a,d).

The median *R*^2^ for all associations that were Bonferroni significant was 0.6% (IQR, 0.3% to 1%; 5th to 95th percentile, 0.1% to 3.6%; Fig. 3b,c). The median *R*^2^ was 0.02% for nonsignificant associations. We observed a range of *R*^2^ by exposure associations across domains of the phenome and exposome (Fig. 3b,c). For example, phenotypes in the inflammation category had exposures that explained 3% of variance on average across all phenotype–exposure associations (Fig. 3b) that were Bonferroni significant. For exposures, pollutant factors explained on average 0–3% of variation across all phenotypes. Organochlorine exposures explained ~3% of variance on average across all phenotypes. On average, dietary biomarkers accounted on average 1% of variation; however, dietary factors measured through an interview explained on average 0.5% of variation in phenotype.

Second, we also estimated the *R*^2^ owing to the additive contribution of 20 exposures simultaneously. For phenotypes that had greater than 20 exposures associated at a FDR level of significance, we imputed exposure data where missing (Methods). When considering 20 exposome factors simultaneously in 119 phenotypes, the median variance explained is 3.5% (IQR, 1.8% to 7.9%), greater than the median *R*^2^ for single exposures (Supplementary Table 6). When considering all phenotypes with ≤20 exposures in the model, the median *R*^2^ was 1.6% (IQR, 0.7% to –3.5%; Fig. 3d, red points).

The maximum multiple exposure *R*^2^ estimated was 43% for triglycerides (Fig. 3d, red points). Triglycerides are an important clinical phenotype used to screen for cardiovascular disease. We found that aggregate exposome, particularly lipophilic dietary and pollutant-related exposures, described large variance in levels of triglycerides in the USA (*R*^2^ of 43%, the largest of any phenotype) (Supplementary Tables 6 and 7), even after adjusting for total cholesterol. Of the 20 variables in the model (Supplementary Table 8), a trans fatty acid (*trans*,*trans*-9,12-octadecadienoic acid), alpha-tocopherol and gamma-tocopherol independently contributed the most to the variance and were positively associated with triglycerides (Supplementary Table 8).

### An atlas across the exposome and phenome

Association sizes correspond to the change in the outcome per 1-s.d. increase in exposure for log-transformed continuous exposures or relative to the reference group for categorical variables (Fig. 4, ‘adjusted beta’). For associations between Bonferroni-significant exposome factors and phenotypes (association sizes for a 1-s.d. change in exposome factor), the 5th to 95th percentile range was −0.17 to 0.19 (0.03 to 0.24 in absolute values).

### Dense correlational web of the exposome

**Fig. 5:**
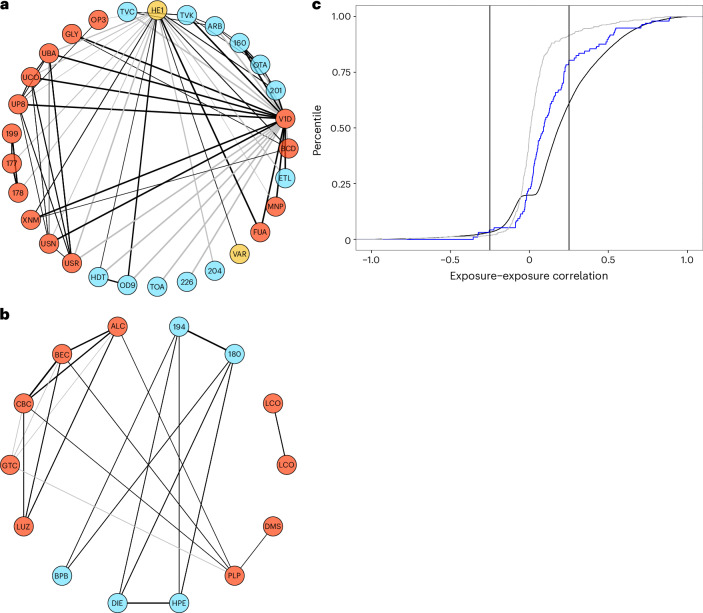
Exposome–exposome correlational globe and distribution of exposure–exposure correlations across the exposome. **a**, An example exposome globe depicts exposure factors and their correlations chosen randomly (thresholded for absolute values of correlations above 0.25). **b**, Exposome correlation globe for exposures associated with hemoglobin A1C or BMI. Node colors include: pollutants (red), infection (yellow) and nutrients (red). **c**, Distribution of exposure–exposure correlations. Gray color denotes randomly selected correlations. Blue line denotes exposome correlations for exposures associated with BMI or hemoglobin A1C. Black denotes correlations that achieved Bonferroni significance. Lines depict correlations at −0.25 and 0.25. Source data

Exposures exhibit a dense correlation web (Fig. 5a,b). The median correlation between exposures was 0.01, and the median absolute value correlation was 0.05 across all correlations. For exposure–exposure correlations that passed the Bonferroni threshold (*P* < 0.05/201,265), the median for Bonferroni-corrected correlations (alpha threshold of 2 × 10^−7^) was 0.19 and the median absolute value correlation was 0.21 (Fig. 5c); the IQR of Bonferroni-corrected correlations was 0.08 to 0.37 (Fig. 5c) (0.11–0.38 for the absolute values). The 95th percentile reached a correlation of 0.69 (0.69 for the absolute values).

The dense correlational web is described for a sampling of exposures in a correlation globe (Fig. 5a,b and Methods). Figure 5a shows 50 randomly selected correlations sampled from all 201,265 correlations. Figure 5b depicts exposures identified in their association with BMI and A1C%. Correlations are only drawn between nodes (or exposures) whose absolute value of correlation was greater than 0.25; these correlations are among the top 15% of the distribution of correlations (Fig. 5c). Correlations for exposures associated with BMI and A1C% overall have larger correlations than a randomly selected subset (Fig. 5c, blue).

### Demographic adjustment influences association sizes

**Extended Data Fig. 3:**
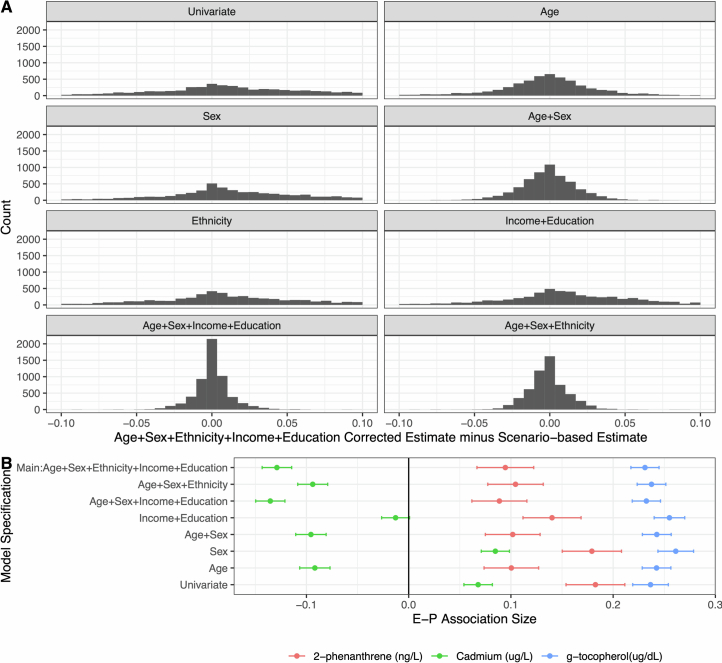
Rate of replication. Percent of total associations identified (at False Discovery Rate of less than 0.05) as a function of the number of surveys available. The rate is divided into associations that could be found in 2, 3, 4, 5, 6, 7, 8, 9, and 10 different and independent surveys. Source data

Next, we computed the difference between adjusted associations (shown in Fig. 4) subtracted from univariate estimates to assess the impact of adjustment. The average difference between a minimally adjusted and a fully adjusted model and each scenario was 0.01 (Extended Data Fig. 3a), demonstrating some bias due to demographic adjustment. The s.d. sizes were the largest for the fully adjusted minus univariate model (s.d. of 0.1) and fully adjusted minus ethnicity model (s.d. of 0.1). The s.d. sizes were the smallest for the fully adjusted minus age, sex and ethnicity models and the fully adjusted minus age, sex and income/education models (Extended Data Fig. 3a).

For some associations, the sign of the association was the opposite depending on the demographic correction scenario. Of the significant findings, 932 out of 5,194 (15% of total significant Bonferroni-identified pairs) exhibited a switch of coefficient sign between the univariate model (a model with no demographic or social factor adjustment) and the adjusted model. For example, BMI and blood cadmium had positive associations (for example, for an increase in exposure—there is linear increase in BMI); however, when controlling or adjusting for factors in the ‘main’ model, the association becomes stronger (for example, the standard errors are reduced) and opposite in direction (Extended Data Fig. 3b). The difference between adjusted associations also differed per exposome domain (Supplementary Fig. 4).

### Consistency of associations across exposure categories

Self-reported dietary nutrients and variables dominate nutrient exposure assessment in epidemiological studies. Overall, we found that 1,452 phenotypes had Bonferroni-significant associations with nutrient variables derived from self-report questionnaires; however, among these phenotypes, they exhibited a median *R*^2^ of only 0.2%.

**Extended Data Fig. 4:**
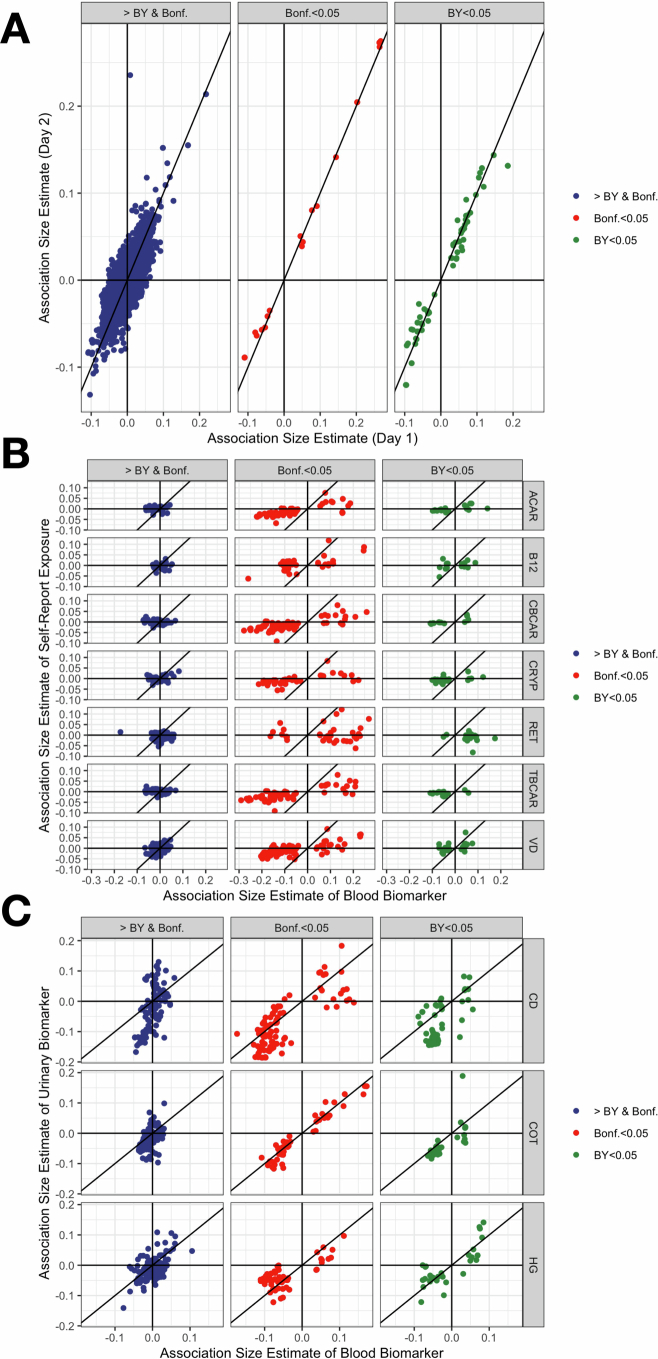
Concordance of phenotype-exposure associations across domains. **A**.) Concordance of PE associations: Day 1 nutrient vs. Day 2. **B**.) Correlation of PE associations for blood vs. self-reported marker of alpha carotene (ACAR), vitamin b12 (B12), cis beta-carotene (CBCAR), cryptoxanthin (CRYP), retinol (RET), trans-beta-carotene (TBCAR), and vitamin D (VD). **C**.) Correlation of PE associations for blood vs. urine biomarker of cadmium (CD), cotinine (COT), and mercury (HG). Source data

Next, we hypothesized that a dilution of correlation size was due to measurement noise. Self-reported dietary nutrients were assessed on 2 days. The median correlation across 69 dietary nutrient recalls on day 1 versus day 2 was 0.36 (IQR, 0.28 to 0.43). We estimated the correlations of associations (for example, the beta carotene–P association on day 1 versus the beta carotene–P association on day 2) (Extended Data Fig. 4a). Across all levels of significance, we observed a 0.84 correlation between day 1 self-report versus day 2 (Extended Data Fig. 4a).

Dietary biomarkers, on the other hand, had a larger median *R*^2^ of 1% across the 1,101 phenotypes with Bonferroni significance, five times larger than their self-reported counterparts. We also estimated the correlation between biomarkers and their self-reported counterparts (for example, day 1/day 2 average of *trans*-beta carotene versus serum *trans*-beta carotene) (Extended Data Fig. 4b). The correlations between the self-reported values and biomarkers were smaller, and we observed a Pearson correlation of 0.52. For those blood nutrient variables that were Bonferroni significant, we observed a correlation of 0.60.

Blood and urine pollutant biomarkers reflect the biological relationship between exposure and excretion. We observed a positive and strong correlation between associations. For example, for blood versus urinary biomarkers, we observed a 0.72 Pearson correlation. When only considering blood biomarkers that were Bonferroni significant, the phenotype–exposure associations between blood versus urine biomarkers was 0.78 for cadmium, 0.96 for cotinine and 0.71 for mercury (Extended Data Fig. 4c).

### Consistency of exposome associations for lung function

**Extended Data Fig. 5:**
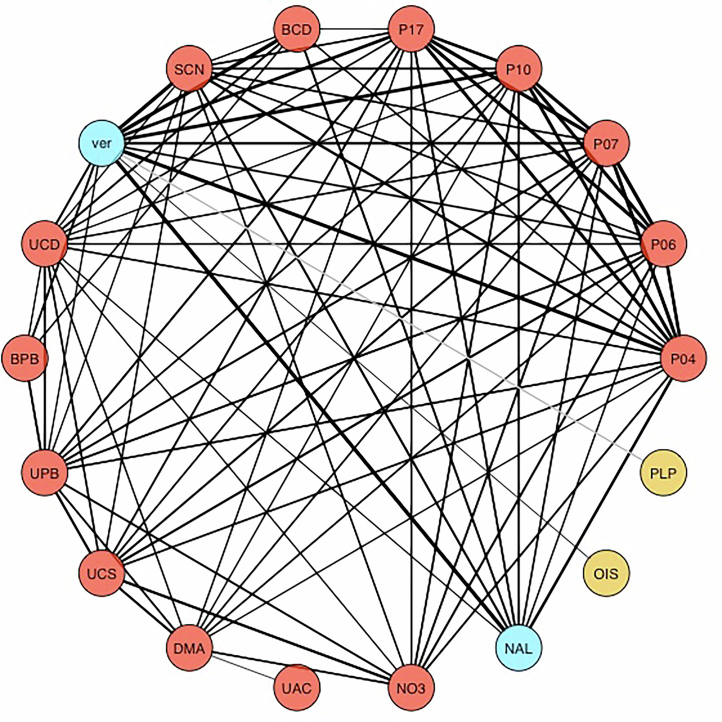
Exposure-exposure correlations for exposures found in association with FEV1. All lines depict a FWER-corrected correlation. NAL: NNAL, urine; OIS: Moisture; UGR: Total sugars; P04: 2-fluorene; P06: 1-phenanthrene; P07: 2-phenanthrene; P10: 1-pyrene; P17: 9-fluorene; BCD: Cadmium; BPB: Lead; NO3: Urinary nitrate; SCN: Urinary thiocyanate; ver: Smoking (current, ever, never); UAC: Urinary Arsenocholine; DMA: Urinary Dimethylarsinic acid; UCD: Cadmium, urine; UCS: Cesium, urine; UPB: Lead, urine; PLP: Pyridoxal 5’-phosphate. Blue color denotes smoking-associated factor, red color denotes pollutant, and yellow denotes dietary factor. Grey line denotes a negative correlation; black line denotes a positive correlation. Source data

Smoking is a strong risk factor for reduction of lung function, such as the amount of air expelled (for example, FEV_1_). Smoking-related biomarkers such as 4-(methylnitrosamino)-1-(3-pyridyl)-1-butanol (NNAL) and serum cotinine indeed show negative associations with FEV_1_, consistent with prior evidence linking tobacco exposure to reduced lung function. Urinary NNAL is a tobacco-specific nitrosamine, which showed a stronger negative association with lung function (FEV_1_ (–0.06 per s.d., *R*^2^ = 0.2%)) compared with serum cotinine (–0.03 per s.d., *R*^2^ = 0.08%). The difference in association between cotinine and NNNL is consistent with their biological properties: cotinine, a short half-life metabolite of nicotine, primarily captures recent exposure and is subject to greater day-to-day variability, whereas NNAL, a metabolite of the tobacco-specific nitrosamine with a half-life of 10–16 days, provides a more stable marker of cumulative exposure. Nevertheless, numerous densely correlated exposures emerged associated with FEV_1_ (Extended Data Fig. 5).

### Exposome correlates of methylation and cognitive aging

We deployed our ExWAS procedure across timely biomarkers of aging, including epigenetic age (for example, Horvath’s biological age predictor and GrimAge (which includes smoking status)^17,18^). We also interrogated the indicators of cognitive recall (digit substitution test), as they are used in the clinic to stage cognitive decline in older adults (Supplementary Fig. 5). Volatile organic compounds (VOCs), smoking indicators (cotinine) and physical activity had the strongest associations with cognitive function (Supplementary Fig. 5a,b). We found shared exposure associations (or, ‘shared architecture’) between better cognitive function and other phenotypes interrogated in the population, including higher exhaled nitrous oxide (shared correlation of 0.35) and with urinary creatinine (Supplementary Fig. 5c).

The strongest signals for accelerated epigenetic age (such as GrimAgeMort) was associated with smoking, heavy metals and physical activity behavior. Among these domains, physical activity explained the most variance (less than 1% in *R*^2^); however, in terms of aggregate total exposomic risk, ten exposures explained 10% of the variance in GrimAgeMort (Supplementary Table 6).

### Age by exposome interactions

**Extended Data Fig. 6:**
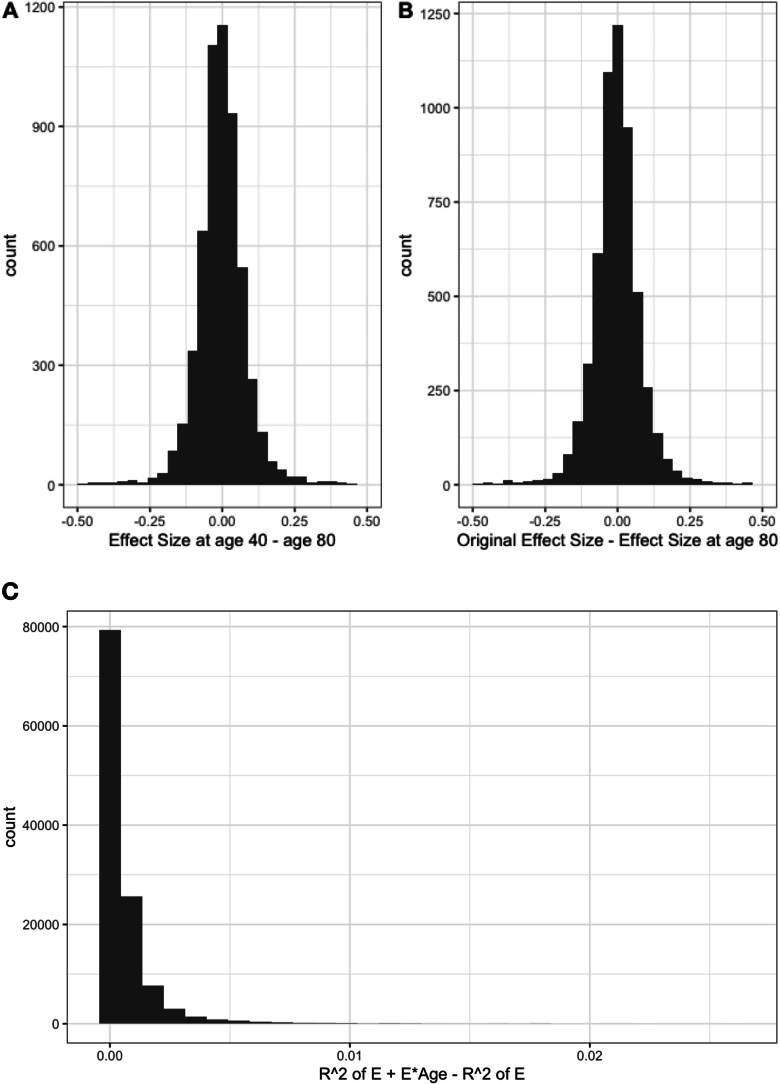
Age-exposome interactions. **A**.) Histogram of effect size estimated at age 40 minus estimated at age 80. Median was -0.0005, IQR: [-0.05, 0.05] **B**.) Original effect size estimate (without interaction term) minus effect size estimate at age 80. Median was -0.00049, IQR: [-0.05, 0.04]. **C**.) Additional variance explained when including an age-exposure interaction term to test for differences in exposure-phenotype associations by age. The median of additional variance explained was 0.0002. Source data

Recognizing that the effect of an exposure may vary depending on an individual’s age at the time of exposure, we modified the ExWAS procedure to consider age by exposure interactions (for example, does the association size differ for individuals at different ages?; Methods). In summary, the inclusion of exposure-by-age interactions resulted in only marginal improvements in the variance explained (*R*^2^) for most phenotypes (Extended Data Fig. 6a–c). Most of the additional variance for models that incorporate an interaction term are limited, although there are exceptions.

### Shared associational architecture between exposures

**Extended Data Fig. 7:**
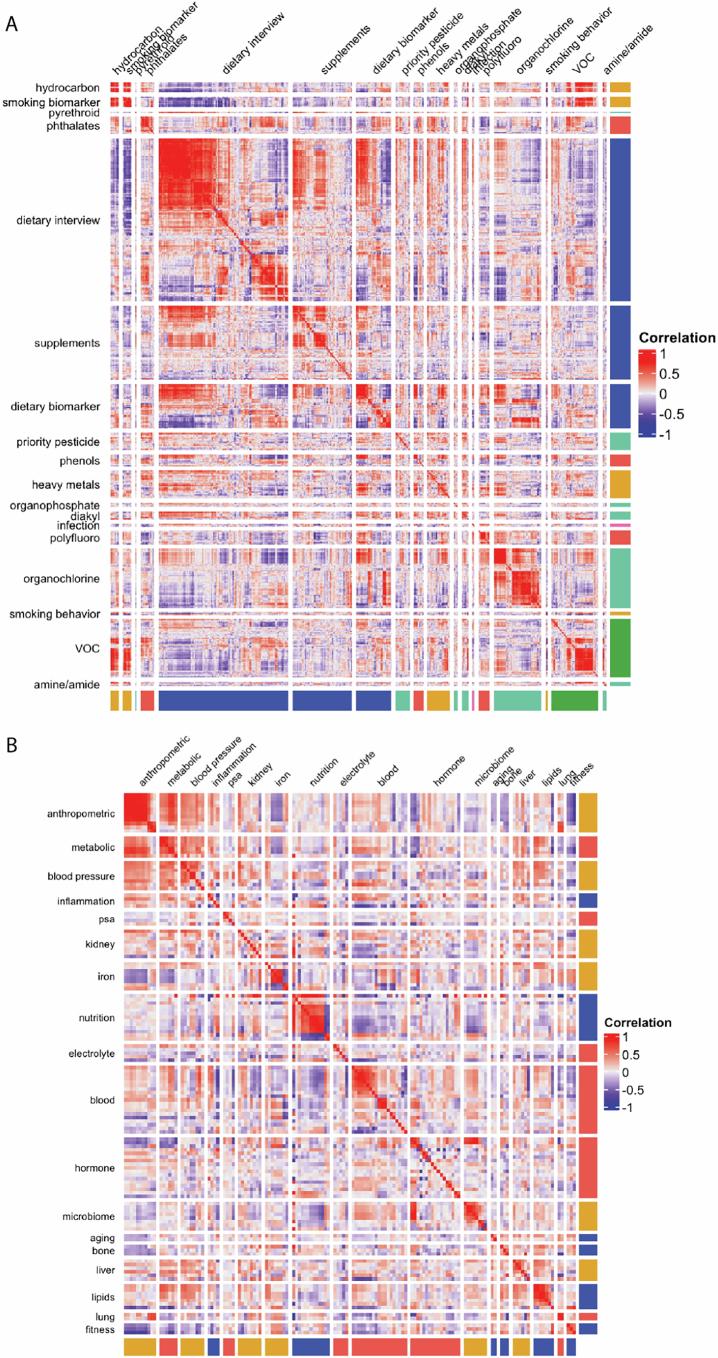
Shared exposomic and phenotypic architecture. **A.)** Shared exposomic architecture for exposures within each domain. (for example, the concordance of beta coefficients across phenotypes). Pairs of exposures that demonstrate more common associations are seen in red; pairs of exposures that demonstrate least common associations are seen in blue). **B**.) Shared phenotypic architecture for exposures within each domain. (for example, the concordance of beta coefficients across phenotypes). Pairs of phenotypes that demonstrate more common associations are seen in red; pairs of phenotypes that demonstrate least common associations are seen in blue). Source data

For a pair of exposures, shared ‘associational architecture’ measures the correlation or the similarity of their correlations across all phenotypes (Extended Data Fig. 7a,b). For example, the correlation between associations for blood *trans*- versus *cis*-beta carotene was 0.98; in other words, the association coefficients across the phenome were very similar for those two exposures. The associational architecture between serum cotinine and 3-fluorene had 0.90 correlation.

Exposures from the same category (for example, dietary interview, organochlorine, VOCs and dietary biomarkers) tended to have similar phenotypic associations; the degree of shared associational architecture is larger within categories than across categories. For all correlations within exposure variables that were dietary biomarkers, the median absolute value of shared architecture was 0.2 (IQR, 0.01 to 0.35). Similar shared associational architecture was observed within smoking biomarkers (0.2, IQR, 0.08 to 0.35). The shared associational architecture between dietary biomarkers and self-reported dietary nutrients had a median absolute value correlation of 0.24. We examined the degree of shared associational architecture between phenotypes. The shared associational architecture between BMI and body weight was 0.98. BMI and cardiorespiratory fitness had opposite associational architecture (the sign of the correlations were opposite between BMI and fitness): a correlation of −0.83. A1C% had an opposite architecture compared with high-density lipoprotein cholesterol (correlation of −0.54).

### Comparison with GWAS

**Extended Data Fig. 8:**
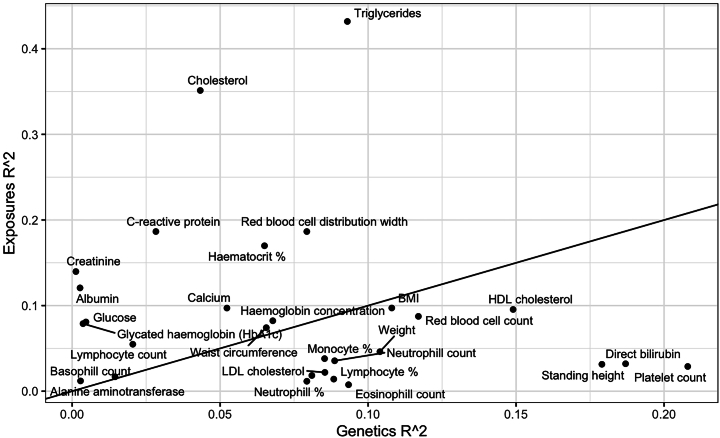
Comparing Exposomic R2 vs. Genomic R2 across 29 quantitative physiological phenotypes. We examined up to 20 exposome factors simultaneously for each phenotype and compared it to the total R^2^ of common genetic variants. Source data

We used data from participants of the UK Biobank to compare genetic versus exposomic predictions. We compared the variance explained due to ~1 M imputed genotypes from genome-wide association studies (GWAS) performed on 29 of the phenotypes^19^ examined here (Extended Data Fig. 8 and Supplementary Table 7). Across the 29 phenotypes, the median incremental *R*^2^ due to genetics was 7.9% (IQR, 2.8% to 9.3%; maximum 21%) and the median incremental *R*^2^ due to exposome (20 exposome variables across 39 phenotypes) was 7.9% (IQR, 3.1% to –12%; maximum 57%). We found that the multiple exposome factors, when modeled simultaneously, had explained variance comparable to the entire genomic array across the phenotypes (Extended Data Fig. 8). Specifically, 55% (*n* = 16) of phenotypes had higher exposomic versus genetic *R*^2^ (Extended Data Fig. 8). For example, the total genetic (1 M common single nucleotide polymorphisms) and exposomic (20 factors) variance explained for BMI was similar, at ~10% for both.

We benchmarked the atlas against three exposure-wide analyses and found that our findings were concordant (directionally consistent and robust *P* values) with previous published data found in refs. ^20–22^.

## Discussion

Our systematic mapping of the exposome onto the phenome reveals three insights with direct clinical and biomedical implications. First, robust environmental signals are highly concentrated in cardiometabolic and pulmonary phenotypes used to stage and gauge care, establishing lipids (specifically triglycerides), glycemic markers and lung function (FEV_1_) as the highest-yield targets for our data-driven environmental risk assessment. Objective nutrient biomarkers and lipophilic pollutants are reproducible correlates of BMI, glycated hemoglobin and lipid profiles. Triglycerides stood out as the phenotype most strongly linked to multidomain exposure patterns, with trans fatty acids, banned persistent pollutants (for example, polychlorinated compounds) and vitamin E isoforms among the most informative contributors, suggesting that lipid risk assessment—important for staging cardiovascular disease—may be sensitive to integrated dietary and pollutant chemical contexts. In pulmonary traits, tobacco-specific biomarkers showed stronger and more stable associations with reduced lung function than short-lived nicotine metabolites, supporting the clinical utility of longer half-life biomarkers when refining smoking-related risk for FEV_1_ and related outcomes (for example, chronic pulmonary disorder). Importantly, we also demonstrate that while single exposures have modest association sizes, aggregate ‘poly-exposomic’ profiles explain phenotypic variance comparable to genome-wide polygenic scores; this suggests that multifactor environmental exposome integration is required to meaningfully improve precision risk models beyond age and sex. Third, we identify a critical reliance on objective measurement: biomarkers (for example, serum nutrients and urinary tobacco metabolites) consistently revealed biomedical associations that self-reported history failed to capture. Collectively, these findings move the field beyond fragmented and nonobjective associations, defining the specific clinical domains and measurement modalities necessary to operationalize the exposome in biomedical research to evaluate medical decision-making.

Our findings have several implications. Estimating association sizes and their replication rate across exposure domains helps to prioritize which exposures are most likely to yield clinically meaningful signals and can guide study design and power planning for ExWAS in new cohorts (Extended Data Table 1). Most of the exposome tabulated here adds little incremental clinically relevant predictive value for many phenotypes, but a smaller set especially in cardiometabolic and smoking-related domains appear more promising for refining risk equations. We note that cancer-related phenotypes are underrepresented in our atlas and are ripe for further research.

Our data suggest that clinically useful environmental risk stratification will more often require integrated, multiexposure models (for example, ref. ^23^) rather than isolated markers. Demographics remain essential for risk adjustment, and modest exposome signal strength probably reflects measurement limitations and the cross-sectional nature of many exposure assessments.

Most exposures show broad, nonspecific associations across phenotypes and are often correlated with other exposures^24–26^, complicating causal interpretation and attribution, underscoring the need to view high-priority signals in the context of exposure ‘mixtures’, globes and bundles. This is exemplified by smoking, where biomarker indicators of the behavior with different half-lives may capture distinct time windows of exposure relevant to lung function; however, the exposures are all related to one another and make a complex globe.

Inferred exposure–phenotype relationships are sensitive to analytical choices and confounding control^27^, especially for age, sex and socioeconomic factors, reinforcing the need for transparent modeling and framing. Future work should test whether top signals persist under alternative adjustment strategies and in longitudinal settings, and the exposome field may need to configure model specifications per exposome or phenotype domain and explore mediation and interaction^28^.

Objective biomarkers appear more consistent and informative than self-reported measures, with strong concordance across blood and urine heavy metal indicators and far weaker signals for dietary recalls compared with nutrient biomarkers. This supports prioritizing standardized biospecimen assays when the goal is clinical translation for precision medicine or robust population surveillance.

We have several technical recommendations for implementation of ExWAS (Supplementary Table 9). Exposomics, at present, is associational discovery. To move toward causal attribution, we recommend a triangulated strategy (for example, ref. ^29^) that prioritizes top exposure–phenotype pairs, ranked by replication rate, effect size, *P* values and vibration of effects^30^ for targeted follow-up. Next, temporality in disease-specific longitudinal cohorts should be established by measuring exposures at baseline and relating them to time-to-event or longitudinal trajectories. One can apply instrumental-variable approaches, including Mendelian randomization^31^, where genetic variants serve as stand-ins for exposures. Third, aim for ‘functional exposomics’ and measure more granularly and precisely, using proteomics, metabolomics and methylomics to map exposure–responsive pathways and test mediation (for example, refs. ^32,33^). For behavioral exposure bundles (for example, diet), investigators should move to randomized interventions.

Our study has limitations, with many directions to evaluate next. Despite cataloging hundreds of factors, we capture only a fraction of the total exposome; characterizing complex exposure–exposure and gene–environment interactions^34^ will require larger sample sizes and broader, high-resolution chemical profiling. While our reliance on objective biomarkers yields stronger signals than the self-reported or geospatial proxies often used in other biobanks, direct cross-cohort comparison is complicated by differences in sampling frames (for example, volunteer bias in cohorts such as UK Biobank^35^). Furthermore, our systematic evaluation suggests that many associations reported in previous candidate–exposure literature may be false positives^9^. The cross-sectional design limits causal inference and the capture of cumulative lifetime exposures. Although we attempted to model participant age as modifying exposure–phenotype relationships, delineating critical windows of susceptibility or nonlinear temporal dynamics ultimately requires longitudinal designs to distinguish chronic accumulation from acute reverse-causal effects. The exposomic architectures measured here are chronic but they may also be acute, such as glucose response to diet and physical activity^36,37^. Frontier studies will incorporate dynamic and personalized exposome and phenotype measurements such as continuous glucose monitoring devices to ascertain heterogeneity or person-specific responses to the exposome^38^.

Our atlas will complement existing global efforts to document exposure–phenotype relationships. For example, there are numerous biobanks and cohorts with phenotype measures, and to some extent, exposure measures such as those documented in the Human Health and Exposure Analysis Resource (https://hhearprogram.org/data-center) (Extended Data Table 1). A recent serum‑only exposome mapping in a Chinese cohort (You et al.)^39^ prioritized breadth and population representativeness, interrogating 267 blood chemicals in 5,700 volunteers. We view our atlas as a complement to these efforts and will help to devise standards by which to catalog associations^40^ to enhance longer-term reproducibility.

Emerging efforts are expanding the molecular exposome with increasingly precise high-resolution assays, including targeted panels and high-resolution mass spectrometry (for example, ref. ^41^), but the next major advance will be enhanced measurement paired with expanded study designs. In particular, repeated and personalized exposure profiling that spans chronic burdens and acute signals will be essential for moving to causal implication of the exposome in disease. Such measurement-rich longitudinal studies, will further prioritize the most promising exposure domains for follow-up, clarify temporality, explain personal heterogeneity and identify modifiable drivers of clinical phenotypes. As these studies mature, the field will be positioned to define causally attributable exposures that can be targeted through behavioral, pharmaceutical, environmental or policy interventions and/or incorporated into predictive models (for example ref. ^42^) for individual risk assessment in the clinic. In this way, our ExWAS serves as a prerequisite for systematic, large-scale integration of exposomic information at the point of care.

## Methods

### Declaration of ethics and consent

This study was deemed ‘not human subjects’ research by the Harvard Institutional Review Board: IRB24-1004. NHANES participants have previously consented their data for use in research, and the protocol has been approved by the National Centers for Health Statistics (NCHS) ethics board: https://www.cdc.gov/nchs/nhanes/about/erb.html.

We systematically associated environmental exposures and phenotypes, called a P-ExWAS^15^, leveraging participant data from the Centers for Disease Control and Prevention (CDC) NHANES. Extended Data Fig. 1 shows the pipeline workflow. To enhance replication, the findings are deployed as a database called a ‘Phenome-Exposome Atlas’ (Supplementary Table 1). Our study complies with the Strengthening the Reporting of Observational Studies in Epidemiology (STROBE) checklist.

We developed an R statistical package (nhanespewas) to conduct all analyses. The features of the nhanespewas includes (1) cataloging of phenotypes and exposures to associate within the NHANES surveys, (2) R package to associate all of the exposures with phenotypes using a survey-weighted linear model^43^ and providing a user-specified array of modeling assumptions (for example, potential confounder and covariate choice), (3) aggregating pairwise associations across independent surveys (a meta-analysis) to output an overall estimate across surveys and assess replicability and (4) providing a browsable database (Phenome-Exposome Atlas) of summary statistics that contain the overall association size or correlation across all survey years interrogated, the standard error of the association, *P* value and variance explained attributable to the exposure (and after consideration of potential adjusting covariates). The code and package is available via GitHub at https://github.com/chiragjp/nhanespewas. An introduction to the package can be found at pe_quickstart.Rmd (https://github.com/chiragjp/nhanespewas/blob/main/pe_quickstart.Rmd).

### Statistics and reproducibility

This study used an observational, cross-sectional design leveraging ten serial waves of the NHANES from 1999 to 2018. No statistical method was used to predetermine sample size. Sample sizes were determined by the availability of participant data within the public-use NHANES database. To ensure robust associational estimates, we limited our analysis to phenotype–exposure pairs with a minimum of 500 participants across at least two survey cycles. Post hoc power calculations were performed to determine the minimum detectable effect sizes (*R*^2^) for the resulting sample ranges (median *n* = 7,464), as detailed below.

No data were excluded from the analyses, except for the prespecified filtering criteria: phenotype–exposure pairs were required to have at least 500 participants and exist in more than one survey wave to be included in the atlas. Participants with missing values for required demographic covariates in a specific model were excluded from that specific regression (complete-case analysis).

As this was an observational study using secondary public health data, the experiments were not randomized and the investigators were not blinded to allocation during experiments and outcome assessment.

Statistical analyses were conducted using survey-weighted linear regression to account for the complex, multistage sampling design of NHANES. Standardized coefficients, *P* values and *R*^2^ were calculated for each pair. Multiple testing was accounted for using both the Bonferroni family-wise error rate (~4 × 10^−7^) and the Benjamini–Yekutieli FDR (<5%). Missing data for multiexposure models were handled via multiple imputation with chained equations. All analyses were performed using R (version 4.x) and the survey package.

To ensure the reproducibility of these findings, the full analytic pipeline is provided as an open-source R package, nhanespewas, available at (GitHub https://github.com/chiragjp/nhanespewas). All summary statistics, including the Phenome–Exposome Atlas, are available in a searchable digital database and archived via figshare.

#### Attaining and cataloging participant data from the NHANES

First, we download all participant data and data dictionaries, encompassing ten surveys (approximately 10,000 total participants per survey) (Extended Data Fig. 1a). Participant data in the NHANES are divided into different tables in five components (demographics, diet, laboratory, questionnaire and examination).

We next categorized each variable downloaded into being a ‘phenotype’ or an ‘exposure’. We defined variables as exposures if they are (1) exogenous in origin (for example, pollutants), (2) biomarkers of exposure (for example, cotinine) or (3) reflective of lifestyle or behavior (for example, self-reported food intake). While many of these variables do exhibit heritability (for example, have genome–environment correlation), it is secondary, as the origin of the factor is still external.

We processed some phenotypic variables and exposure variables. Blood pressure was a phenotypic variable whose measurement was repeated multiple times: we took the average of the measurements. Physical activity questionnaire information was collected in a series of questionnaire items; we processed these measurements to be in total metabolic equivalent hours (see websites in refs. ^44–46^). We processed multiple smoking variables (see ref. ^26^) to be a variable that denotes past, current or never smoking. We next tabulated the sample size for each exposure and phenotype variable pair used in downstream pipelines.

We programmatically ingested NHANES data dictionaries and public data files for ten cycles (1999–2018) into a SQLite database and built a canonical catalog that maps each exposure (E) and phenotype (P) to all cycle-specific variable names and labels referring to the same construct (as above). The code paths were: download/ (data & dictionary retrieval) and select/ (catalog building, sample-size tables). Tutorials (pe_quickstart.Rmd, pe_catalog.Rmd) illustrate end-to-end usage. The repository and compiled database links are provided in the ‘Data availability’ and ‘Code availability’ sections. When the NHANES supplies both conventional and International System of Units (SI) variables (for example, LBD*SI), we converted to a common unit before transformation; for categorical variables whose levels changed across cycles (for example, education), we recoded to harmonized bins listed in the catalog (Supplementary Tables 2 and 3and Methods). For analytes with known method revisions, we preferentially used NHANES-standardized variables when available—for example, liquid chromatography–tandem mass spectrometry (LC–MS/MS)-standardized 25-hydroxy-vitamin D (LBXVIDMS/LBDVIDMS; bridging equations applied by the CDC) and isotope dilution mass spectrometry (IDMS)-standardized serum creatinine—so that pre- and postchange data are comparable on a common scale. For laboratory measures, the NHANES provides per-analyte lower limit of detection (LLOD) and an ‘…LC’ comment code. Values below the LLOD are set to LLOD/√2, and the proportion <LLOD is tracked per analyte and cycle to aid interpretation and sensitivity analyses. Day 1 and day 2 dietary recalls are treated as distinct exposures; we present concordance analyses of P–E coefficients across days and (where available) compare interview-based nutrients with their biomarker counterpart. This avoids conflating changes in the underlying United States Department of Agriculture nutrient databases with exposure measurement error. For vitamin D, we used the CDC’s LC–MS/MS-standardized variables (LBXVIDMS/LBDVIDMS) across waves to harmonize measurements from earlier radioimmunoassay methods to LC–MS/MS equivalence. For creatinine, we used NHANES IDMS-standardized serum creatinine (and NHANES-recommended corrections for older cycles where applicable). These steps follow CDC/NCHS analytic notes.

To ease interpretation, we categorized each cataloged phenotype or exposure variable into different categories. The categories of phenotypic variables (*n* = 305) that included anthropometric (*n* = 11, for example, height), aging (*n* = 29, for example, telomere length and epigenetic aging), blood parameters (*n* = 19, for example, basophils number), bone (*n* = 3, total calcium), dual-energy X-ray absorptiometry (DEXA) (*n* = 136, for example lean fat), electrolyte (*n* = 5, for example, bicarbonate), fitness (*n* = 3, for example, VO_2_max), hormone (*n* = 17, for example, iodine), inflammation (*n* = 4, for example, CRP), iron (*n* = 8, for example, iron), kidney (*n* = 8, for example, urinary creatinine), lipids (*n* = 7, for example, apolipoprotein B), metabolic (*n* = 6, for example, glucose), microbiome (*n* = 16, for example, observed operational taxonomical unit mean), nutritional status (*n* = 13, for example, folate) and prostate-specific antigen (PSA; *n* = 4, for example, PSA ratio).

The categories of exposome variables (total *n* = 619) included VOCs (*n* = 64, for example, nitromethane), amine/amide (*n* = 5, for example, acrylamide), diakyl (*n* = 9, for example, dimethylphosphate), dietary biomarkers (*n* = 49, for example, vitamin B12), nutrients from dietary interview (*n* = 178, for example, alpha carotene), heavy metals (for example, blood lead), hydrocarbons (for example, 1-napthol), infection (*n* = 31, for example, urinary chlamydia), organochlorine (*n* = 65, for example, PCB199), organophosphate (*n* = 5, for example, acephate), phenols (*n* = 13, for example, urinary *tert*-phenol), phthalates (n = 19, for example, mono(carboxynoyl) phthalate), polyfluorinated compounds (n = 15, for example, 2-(*N*-methyl-perfluorooctane sulfonamido) acetic acid), priority pesticides (*n* = 19, for example, 2,4,5-trichlorophenol (ug l^−^)), pyrethoid (*n* = 1, for example, oxypyrimidine), smoking behavior (*n* = 9, for example, are you a current, ever or never smoker?), smoking biomarker (*n* = 12, for example, cotinine) and supplements (*n* = 83, for example, number of supplements reported). The entire list is presented in Supplementary Tables 2 and 3.

#### Model assumptions and data processing

The core functions of the package we now describe are available in quantpe.R and petable.R (Extended Data Fig. 1a). We model the relationship between an exposure and phenotype pair in the population with the following linear model:

*P*_p_ = intercept + *b*_i _× *E*_i_ + error, where *E*_i_ is an individual environmental factor or exposure of the exposome, the intercept is the average value of the phenotype when the exposure value is 0, and the *b* term denotes the association between the exposure and the phenotype. The lowercase p and i indexes a specific phenotype and exposure, respectively. We assume the error is also normally distributed with a 0 mean and s.d. of 1. We modify this core model in a few ways to consider multiple adjustment scenarios and transformation of the *P*_p_ and *E*_i_ variables.

We transform the exposure and phenotype variables to ensure the comparability of estimates (for example, *b*_i_) across all pairwise phenotype–exposure associations (Extended Data Fig. 1b). Our pipeline inputs phenotypes, such as weight, BMI and glucose, are quantitative and continuous valued variables. All phenotype variables are ‘scaled’ (mean subtracted and divided by the survey-weighted s.d.). Our pipeline also permits rank-based inverse normal transform, commonly used in GWAS^47^.

We transform the exposures (*E*_i_) in the following ways:

1. For continuous and blood or urine biomarkers of exposure, we log transform (base 10) and scale (mean subtracted and divided by the survey-weighted weighted standard deviation). To account for zero values before log_10_ transformation, we add a small constant equal to the smallest nonzero value
2. For categorical exposure variables, we chose the largest category as the reference group and analyzed the categorical variable in the same model
3. For ordinal variables, we kept as ordinals and analyzed as a numbered rank (for example, 1–3)

#### A pipeline to conduct survey-weighted associations

The NHANES is a complex and multistage cross-sectional survey designed by the NCHS; the multistage survey design provides US-wide generalizability for phenotype–exposure associations. Because of the complex study design, unique analytics approaches must be used to ensure accurate association mapping between exposure and phenotype (versus approaches that assume a simple random sample). The NHANES is sampled hierarchically: the first stage of sampling consists of regions of the country or primary sampling unit (PSU) that are selected proportional to the average age, sex group prevalence, race and income. The second stage of sampling includes segments and households; segments within the PSUs that are consistent with census blocks. The third stage of sampling includes individual participants. Each participant is also a weighted sample, such that it is proportional to the number of people in the population that that person represents by age, race, income and nonresponse rate.

Our pipeline iterated through all combinations of phenotypes and exposures in the NHANES:

1 [surveys, phenotypes, exposures] = select_phenotype_and_exposure_pairs (phenotypes, exposures)

2For each Pp [phenotypes]:

3For each Ei [exposures]:

surveys = get_surveys_for_pheno_expo(Pp, Ei)

5data = get_expo_pheno_tables(Pp, Ei, surveys)

6weighted_data = figure_out_weight_across_surveys(data)

7weighted_data = transform_exposure_phenotype(weighted_data)

8weighted_data = create_survey_design(weighted_data)

9model = run_model(Pp, Ei, covariates, weighted_data)

10stats = summary_stats(model)

The NHANES participant data are organized into multiple tables on the basis of properties of the measures that include the type of measure (for example, dietary indicator or biomarker of exposure) on a subsample of the overall sample (Extended Data Fig. 1a). Specifically, demographic variables, such as age, sex, race/ethnicity and income are measured on the overall and entire sample; however, some assays of the exposome and phenome are measured on only a subsample. A tutorial on the ExWAS approach in R can be found here: https://github.com/chiragjp/nhanespewas/blob/main/exwas_tour.Rmd

First, in line 1, we select pairs of phenotypes and exposures that have at least 500 measured participants. We further only select pairs that are present in more than one survey sampling. In lines 5–10 (Extended Data Fig. 1b–d), we developed functions that query and merge the three or more data tables that contain the (1) phenotype, (2) exposure variables and (3) demographic variables and calculate the subsample weights to create a new NHANES-specific data table structure (see functions get_expo_pheno_tables and figure_out_weight in the package). Next, we needed to combine data across multiple survey samplings and compute the new sample weights. For each survey, we used the subsample weights that corresponded to the smallest subsample being analyzed. For example, to estimate an association between fasting glucose (*P*) and urinary phthalate (*E*) biomarker, the data table that contains the smallest sample size will be the sample weight chosen. Last, we averaged the survey weights in accordance with the guidance provided by the NCHS^48^.

NHANES public-use data do not include state/county geocodes. All analyses used the survey R package with the provided PSU and strata identifiers and the appropriate exam/subsample weights for each exposure–phenotype pair. This approach yields design-consistent estimates that are generalizable to the US noninstitutionalized population and absorbs regional composition via poststratification.

Before finalizing covariates, we examined the age–phenotype relationship using nonparametric smooths on representative phenotypes across categories (anthropometry, kidney, lipids and inflammation). We observed curvature; consequently, all models include age and age^2^ to capture low-order nonlinearity with minimal complexity.

#### Cohort characteristics, sample size and power

Each exposure–phenotype pair can be assessed in multiple surveys (1–10) and/or have multiple categorical levels (for example, smoking is a categorical variable with two levels and a reference group).

We considered only phenotype–exposure pairs that had at least 500 participants across two or more surveys resulting in 278 unique phenotypes and 619 unique exposures with complete cases across all possible demographic covariates. The total number of associations possible to assess in 2, 3, 4, 5, 6, 7, 8, 9 and 10 independent surveys was 34,206, 21,451, 34,048, 9,011, 8,412, 1,702, 9,741, 999 and 1,455, respectively. To maximize power, for each association, we combined across two or more surveys, combining the sample weights as advised by the NCHS (Methods).

The range of the sample sizes per association included 608–68,495 individuals. The median sample size was 7,464, with an IQR of 4,220 to 15,316. For a sample size of 608, the lowest *R*^2^ that can be identified was 0.05 with a power of 80% and a *P* value threshold of 1 × 10^−6^. For the same power and *P* value threshold, for sample size of 4,200, the lowest *R*^2^ that can be identified was 0.007; for a sample size of 7,464, the lowest *R*^2^ that can be identified was 0.004; for a sample size of 15,316, the lowest *R*^2^ that could be identified was 0.002; and for the maximum sample size (68,315), the lowest *R*^2^ that could be identified was 0.001.

### Statistical analysis

We cataloged a total of 322 real-valued phenotypes and 859 biomarkers or self-report questionnaire responses that measure environmental, dietary or behavioral exposures across all ten surveys. Each exposure–phenotype pair can be assessed in multiple surveys (1–10) and/or have multiple categorical levels (for example, smoking is a categorical variable with two levels and a reference group). Across 322 phenotypes, 859 exposures, 10 surveys and multiple variable levels, the total number of possible associations were 563,627. We present recommendations for ExWAS analytics in Supplementary Table 9.

In our pipeline to maximize power and replication, we filtered for phenotype–exposures pairs that had at least 500–600 participants across at least two or more surveys (maximally, a phenotype and exposure would appear in all ten surveys), resulting in 305 unique phenotypes and 619 unique exposures. We calculated 119,643 associations between a phenotype (for example, BMI) and exposure (for example, beta carotene) pair. The total number of associations in 2, 3, 4, 5, 6, 7, 8, 9 and 10 independent surveys was 34,206, 21,451, 34,048, 9,011, 8,412, 1,702, 9,741, 999 and 1,455. We used survey-weighted regression to associate phenotypes with exposures while adjusting for age, age^2^, sex, race/ethnicity, income, survey year and education. We also performed associations with no adjustments (univariate) in each survey separately.

We associated *P* (total of 305) with every *E* (total of 651) in which overlapping samples were available across the surveys (ten total surveys; 1999–2000, 2001–2002, 2003–2004, 2005–2006, 2007–2008, 2009–2010, 2011–2012, 2013–2014, 2015–2016 and 2017–2018). An association could occur in 1, 2, 3, 4, 5, 6, 7, 8, 9 or 10 surveys. We used survey-weighted regression to accommodate complex survey design, including PSU, stratum and subsample weights as input and implemented in ref. ^49^ (Extended Data Fig. 1c,d). We considered two models to account for demographic-based confounding or stratification, a base model (as above) and a ‘demographic-adjusted’ model, which consists of covariates age, age^2^, race/ethnicity (non-Hispanic white (reference), non-Hispanic Black, Mexican American, other Hispanic and other ethnicity), household-to-income poverty ratio (ranging from 1 to 5), education (less than ninth grade, less than high school, high school graduate (reference), attended college and college graduate) and survey as a categorical variable. The summary statistics consists of the association sizes (beta coefficients), standard errors, *P* values, the sample size, surveys and *R*^2^ for the entire model.

For test statistic and *P* value estimation, we report ‘robust’ standard errors implemented in the survey package. We estimated the Bonferroni family-wise error rate and the Benjamini–Yekutieli FDR^50^ across all tests and primarily report findings that exceed the family-wise error rate (Bonferroni). We also secondarily report the FDR at thresholds less than 5%. We also estimate the ‘exposome inflation factor’^51^.

We also estimated concordance of phenotype–exposure associations across independent survey samplings (Extended Data Fig. 1d,e). First, we calculated the variation of the phenotype–exposure associations across independent survey samples. For example, if a phenotype–exposure association was measured in three surveys, we estimated the association separately for each survey and calculated the variation of the phenotype–exposure correlation across the three surveys. We used an ‘unrestricted weighted least squares’ meta-analytic approach^52^ to estimate cross-survey standard error and heterogeneity estimates (degree to which variation on association sizes can be explained across surveys, for example *I*^2^).

Separate and independent samplings of the US NHANES by year (for example, 1999–2000 or 2003–2004) provide an opportunity to estimate ‘replication’ rates or the times that a phenotype–exposure association has a lower nominal significance level in greater than one survey sample. An association can occur in two to ten surveys. For each scenario, we counted the number of times a phenotype–exposure association was achieved with *P* value less than 0.05; for example, for a phenotype–exposure association that can be assessed in five surveys, that phenotype–exposure association can be significant up to five times.

#### Exposure–exposure correlation globes

We measured the partial Pearson correlation between 619 exposures with pairwise complete data, for a total of 200k correlations. The partial correlation is the correlation between exposures, adjusted by age, age^2^, income-to-poverty threshold, education and ethnicity. To visualize an ‘exposome globe’ as an example, we queried the correlation database for correlations among randomly selected exposures and exposures associated with BMI or glycated hemoglobin. We filtered to only visualize correlations that were greater than 0.25. We visualized the globe using the igraph R package^53^.

#### Shared associations between exposures and phenotypes

We defined the ‘shared’ associational architecture between pairs of exposures (for example, phthalate and heavy metal) or phenotypes (for example, BMI and glucose) by the similarity of their phenotype–exposure associations. Specifically, for a given exposure, there is an array of associations estimated for *X* number of phenotypes (for example, a row in the Atlas; Fig. 3). We estimate the correlation distance between a pair of exposures. For example, if a pair of exposures (for example, *E*_1_ and *E*_2_), have the same association for each n phenotypes (for example, *P*_1_… *P*_n_), their association architecture is equal and the correlation will be 1.

#### Estimating the aggregate contributions of genetic and exposomic factors to phenotypes

To compare the variance explained by exposomic and genetic factors across 29 phenotypes, we constructed and evaluated multivariate models using a three-step workflow. First, for exposome-based modeling, we identified exposures significantly associated (FDR-adjusted *P* value <0.05) with each phenotype using univariate regressions. Among these, we selected up to the top 20 exposures on the bases of their univariate *R*^2^ contributions. We multiple imputed missing data using multiple imputation with chained equations^54^, generating ten imputed datasets via predictive mean matching. For each phenotype, we fit two linear regression models: a baseline model incorporating demographic covariates (age, sex, ethnicity and education) and an expanded model that additionally included the selected exposures. The incremental *R*^2^ attributable to exposures was calculated as the difference in variance explained between these two models.

Second, for genetic modeling, we leveraged previously published GWAS summary statistics derived from approximately 1 million genetic variants (UK Biobank) across 125 quantitative phenotypes of anthropometry and biomarkers. A total of 29 of these 125 phenotypes (23%) overlapped with those queried in this investigation. Genetic contributions (*R*^2^) to each phenotype were obtained from quantitative trait GWAS models that included age, sex, genotyping array and principal components as covariates. The genetic *R*^2^ was defined as the incremental variance explained by the genetic model beyond these baseline covariates. The median genetic *R*^2^ across all 125 phenotypes queried as reported in ref. ^19^ was 7% (IQR, 4% to 9%; max *R*^2^ of 36%).

We directly compared exposome-based and genetic-based *R*^2^ values across the 29 overlapping phenotypes (Supplementary Table 4). We selected phenotypes on the basis of the availability of matched phenotype data from the NHANES and the UK Biobank. This allowed assessment of the relative explanatory power of exposomic versus genetic factors for each phenotype, critically informing the conclusions about the comparative utility of exposomic data relative to genetic predictors.

#### Concordance of associations with different types of measurements of the same exposure

Some variables are assessed at different times (part of a 2-day interview) or sample matrices (for example, in both urine and blood). We examined the concordance of phenotype–exposure associations across these samplings (for example, day 1 exposure versus day 2 or blood versus urine measure). For self-reported nutrients, we compared the phenotype–exposure associations for day 1 versus day 2: the number of dietary supplements reported, calcium, carbohydrate, copper, folic acid, folate, dietary folate equivalents, iron, energy, lycopene, lutein + zeaxanthin, magnesium, niacin, phosphorus, potassium, selenium, thiamin (vitamin B1), vitamin B12, riboflavin (vitamin B2), vitamin B6, vitamin C, vitamin D (D2 + D3), vitamin K, zinc, total fat, iodine and total sugars. We also compared the phenotype–exposure associations between self-reported measures and their biomarker counterparts, including alpha carotene, vitamin B12, *cis*-beta carotene, cryptoxanthin, retinol, *trans*-beta carotene and vitamin D. Last, we compared phenotype–exposure associations between blood-based and urine-based indicators, including mercury, cadmium and cotinine.

### Reporting summary

Further information on research design is available in the Nature Portfolio Reporting Summary linked to this article.

## Online content

Any methods, additional references, Nature Portfolio reporting summaries, source data, extended data, supplementary information, acknowledgements, peer review information; details of author contributions and competing interests; and statements of data and code availability are available at 10.1038/s41591-026-04266-0.

## Supplementary information

Supplementary Information Supplementary Figs. 1–5.

Reporting Summary

Supplementary Tables 1–9 Supplementary Table 1. Summary statistics for all 100k *E*–*P* associations. The column evarname is the NHANES variable name for the exposure, and pvarname is the NHANES variable name for the phenotype. The field estimate.uwls reports the unrestricted weighted least-squares (uwls) meta-analytic beta coefficient pooled across all contributing survey waves, and std.error.uwls is its corresponding standard error. The significance of the pooled coefficient is given by p.value.uwls, while statistic.uwls shows the test statistic (*Z* or *t*) associated with that meta-analysis. Heterogeneity metrics include Q.uwls, which is Cochran’s *Q* statistic, h.squared.uwls, the *H*^2^ heterogeneity ratio, and i.squared.uwls, the *I*^2^ percentage of total variation attributed to heterogeneity; k.uwls denotes the number of independent NHANES survey waves (*k*) used. To assess consistency across cycles, model_concordance counts how many survey waves achieved a nominal *P* value <0.05, and model_concordance_1_2 counts how many waves had *P* < 0.05 with an effect sign matching the overall estimate. The fields model_number and total_n indicate, respectively, which numbered the adjustment scenario (covariate set) was used in the regression and the total sample size summed over all included waves. Two columns, rsq_adjusted_base_diff and rsq_adjusted_diff, report differences in *R*^2^: the former is the change in explained variance between the fully adjusted and unadjusted (base) models, while the latter is the change between the fully adjusted model and the next less-adjusted model in sequence. For multiple testing correction, pval_BY gives the Benjamini–Yekutieli false discovery rate-adjusted *P* value across all tests, and pvalue_bonferroni gives the Bonferroni-corrected *P* value. The categorical field sig_levels provides a symbolic significance indicator on the basis of those corrected *P* values. Descriptive labels for each phenotype include pvardesc and its broad pcategory and psubcategory, and similarly evardesc, ecategory and esubcategory describe each exposure. In addition, the fields pnewsubcategory and enewsubcategory represent any further refined subcategory assignments made programmatically. Finally, the super-category of each exposure is denoted by super_ecat_number, a numeric code, and super_ecat_name, its corresponding text label. Supplementary Table 2. Exposome Catalog. Evarname is the NHANES variable identifier, super_ecat_name is the high level category name, enewsubcategory is the category of the variable and evardesc is the description of the variable. Supplementary Table 3. Phenotype Catalog. Pvarname is the NHANES variable identifier, pcategory is the phenotype category and pvardesc is a description of the variable. Supplementary Table 4. Exposure categories. *N*, number of variables in category. Supplementary Table 5. Phenotype categories. *N*, number of variables in category. Supplementary Table 6. *R*^2^ for each phenotype and the number of exposures in the model. Supplementary Table 7. *R*^2^ for genetics and exposome for 29 phenotypes. Supplementary Table 8. Coefficients, standard errors and *P* values for multiple exposures (multivariate model) associated with triglyceride levels. Supplementary Table 9. Technical recommendations for implementing an exposome-wide study.

## Source data

Source Data Fig. 2 Statistical source data and axis labels. Source Data Fig. 3 Statistical source data and axis labels. Source Data Fig. 4 Statistical source data. Source Data Fig. 5 Statistical source data. Source Data Extended Data Fig. 2 Statistical source count data. Source Data Extended Data Fig. 3 Statistical source data. Source Data Extended Data Fig. 4 Statistical source data. Source Data Extended Data Fig. 5 Statistical source data. Source Data Extended Data Fig. 6 Statistical source data. Source Data Extended Data Fig. 7 Statistical source data. Source Data Extended Data Fig. 8 Statistical Source data.

## Figures and Tables

**Extended Data Table 1:**
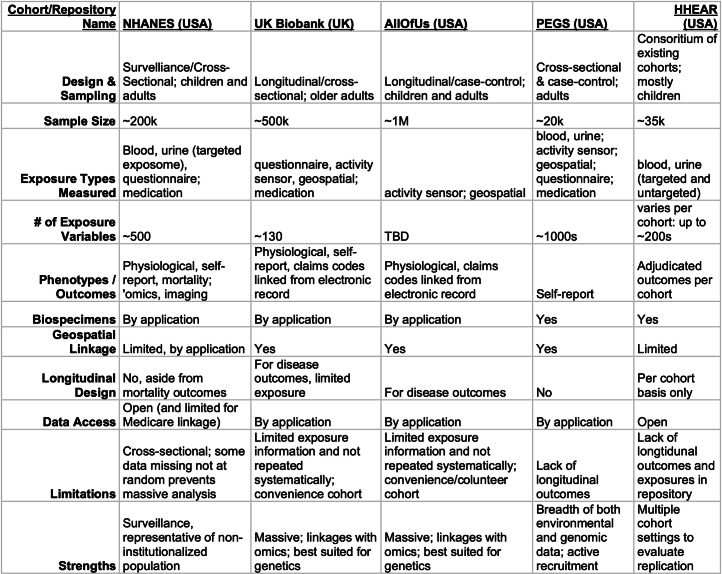
Examples of cohorts

Examples of cohorts (National Health and Nutrition Examination Survey [NHANES], UK Biobank, US AllOfUs, Human Health Exposure Analytics Resource [HHEAR], Personalized Genes and Environment Study [PEGS]) for exposome-wide study.

## Data Availability

The full cohort database can be found at 10.6084/m9.figshare.29182196. The full summary statistics can be found at 10.6084/m9.figshare.29186171. NHANES data are publicly available: https://wwwn.cdc.gov/nchs/nhanes/default.aspx. All results from the PE-WAS can be visualized, downloaded and browsed online: https://pe.exposomeatlas.com. Specifically, users may query an ExWAS for a specific phenotype and examine phenotypic specific associations, *R*^2^ and exposome correlation globes. Source data are provided with this paper.

## Code availability

All code is available as an R analytics package developed under an MIT license via GitHub at https://github.com/chiragjp/nhanespewas. Analytic code was written under R version 4.4.2. The libraries required for the package include DBI, RSQLite, dplyr, tidyr, survey, stats, tibble, broom, rlang, tidyselect, logger, purrr, stringr and ggplot.
