# Supplementary material for: An atlas of exposome–phenome associations in health and disease risk
Source: Nat Med. 2026 Mar 18;32(4):1501–10. doi: 10.1038/s41591-026-04266-0 (PMC13099396; doi:10.1038/s41591-026-04266-0)
Supplement: Supplementary file 1 — Supplementary Figs. 1–5. [file 41591_2026_4266_MOESM1_ESM.pdf]

nature medicine

Supplementary information https://doi.org/10.1038/s41591-026-04266-0

An atlas of exposome–phenome associations in health and disease risk

In the format provided by the authors and unedited

Supplementary Figures

A

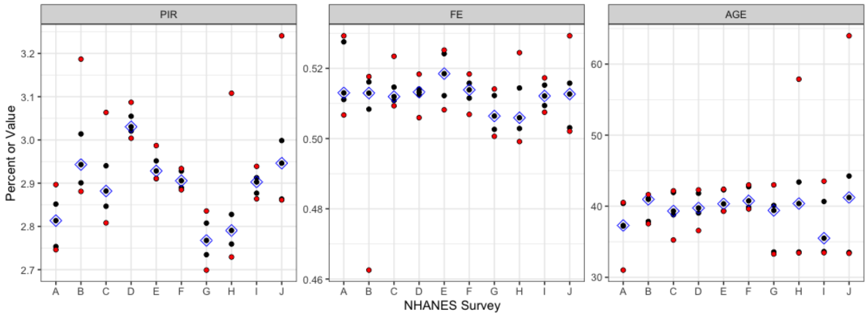

B

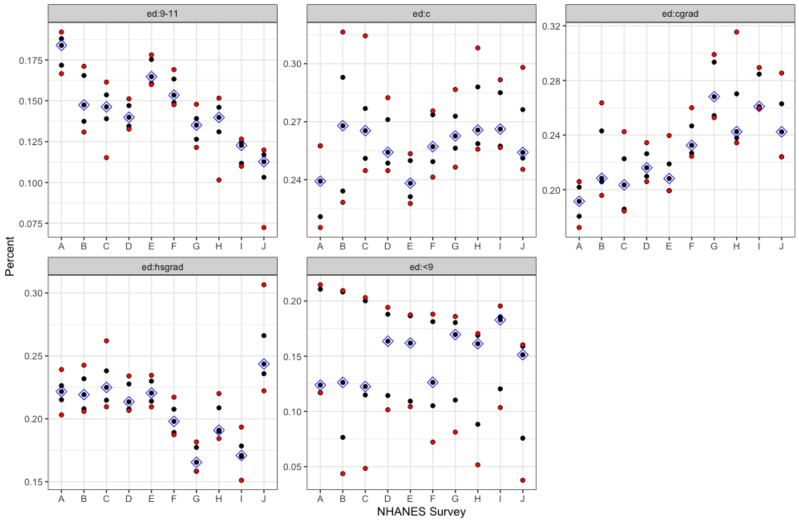

C

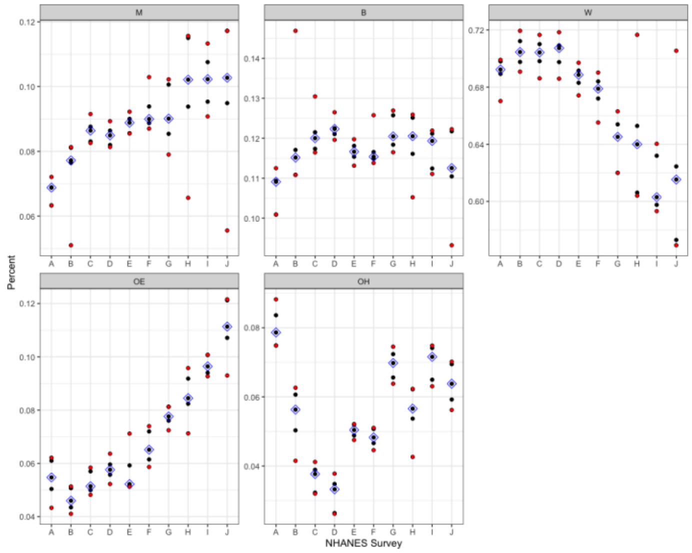

Figure S1. A.) Distribution of income, percent female, and age for each P-E association per survey and variable. Blue diamond denotes the median; the red points denote the 10th and 90th percentiles; black points are the 25th and 75th percentiles. Variables include PIR: Household Income to Poverty Ratio [ranges from 1-5]; FE - female percent. Letters A-J denote survey period (e.g., A: 1999-2000, B: 2001-2002, etc). B.) Distribution of education for each P-E association per survey and variable. Blue diamond denotes the median; the red points denote the 10th and 90th percentiles. Variables include PIR: Household Income to Poverty Ratio [ranges from 1-5]. Ed:9-11- education achieved between 9-11; ed:c - attended college; ed:cgrad - graduated college; ed:hsgrad - graduated high school; ed:<9 - less than 9th grade. Letters A-J denote survey period (e.g., A: 1999-2000, B: 2001-2002, etc). C.) Distribution of Ethnicity for each P-E association. M - Mexican; B - Non-Hispanic Black; W - Non-Hispanic White; OE - Other Ethnicity. Letters A-J denote survey period (e.g., A: 1999-2000, B: 2001-2002, etc).

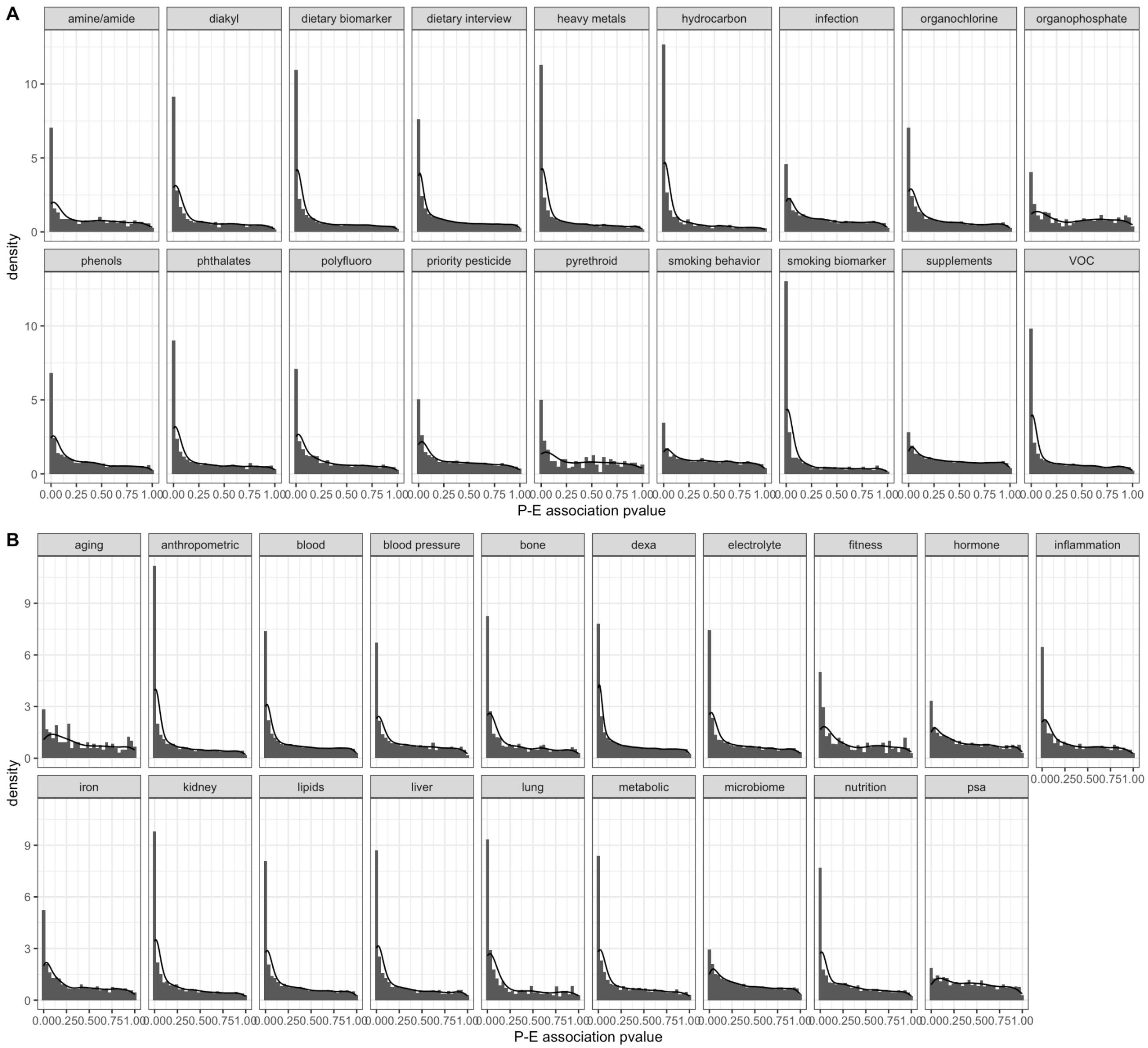

Figure S2. P-value distribution for PE associations, binned by exposome categories (A) and phenotype categories (B).

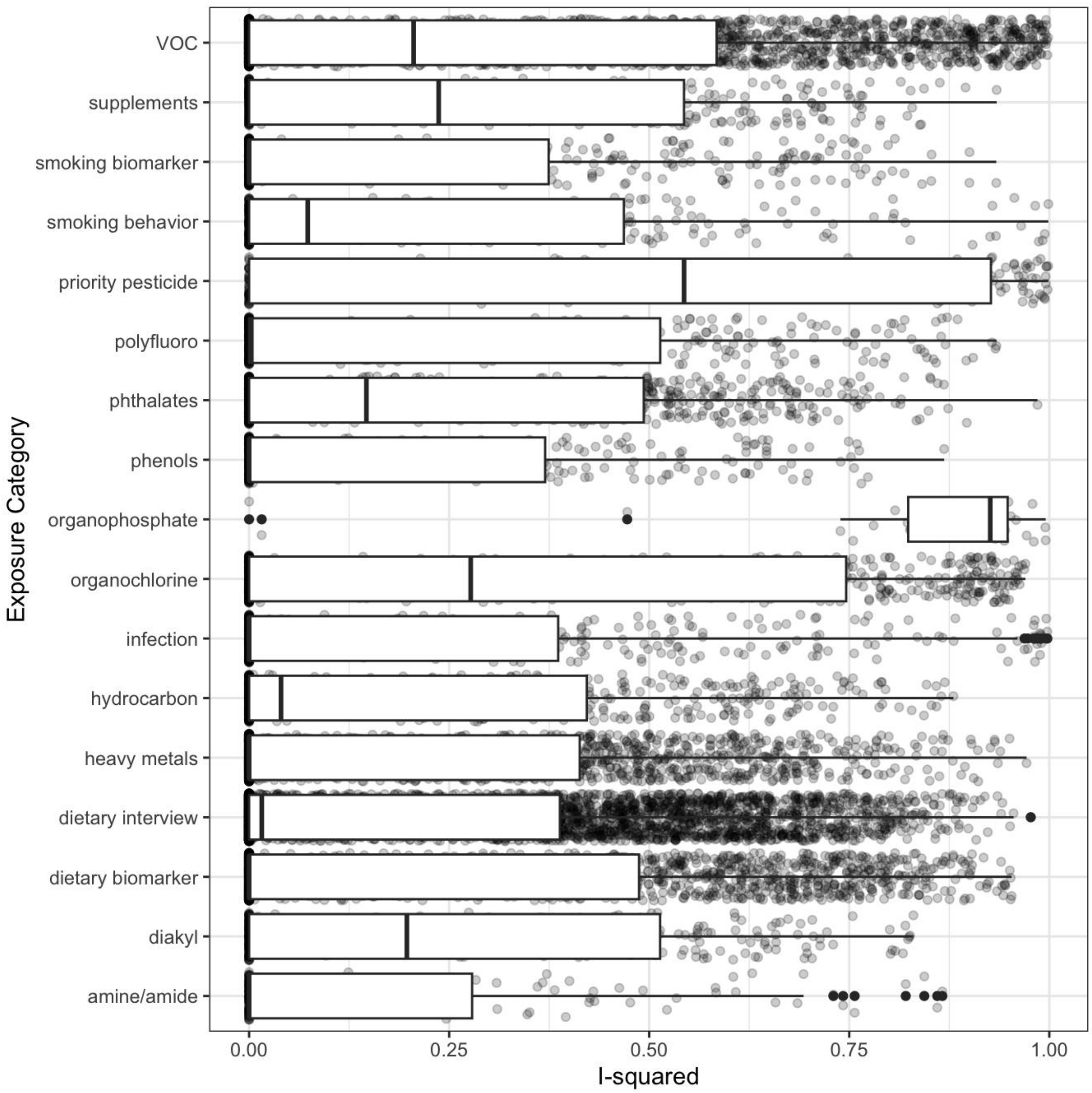

Figure S3. I^2 of P-E associations for exposure categories.

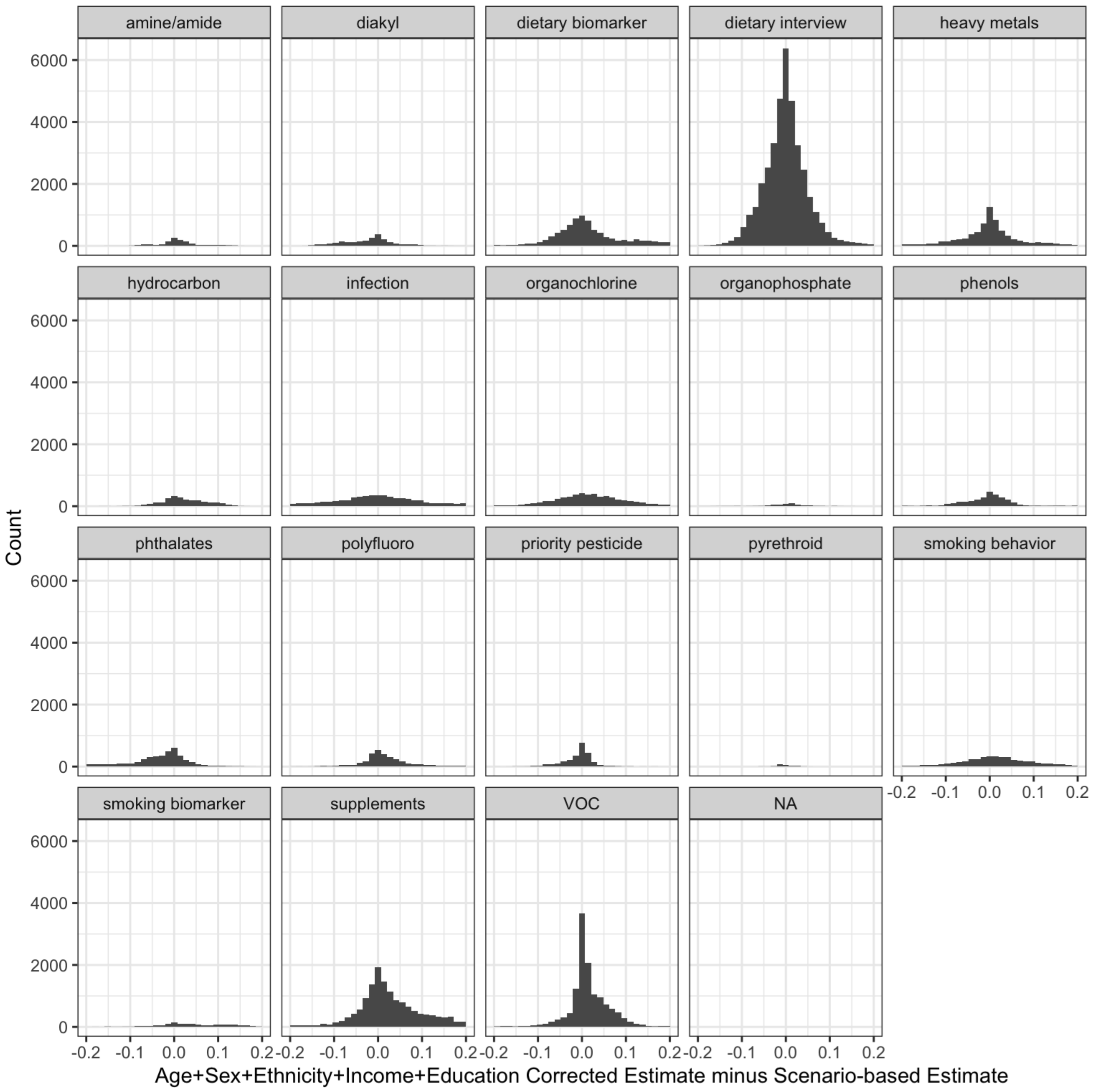

Figure S4. Distribution of fully adjusted minus minimally adjusted models across different exposure categories.

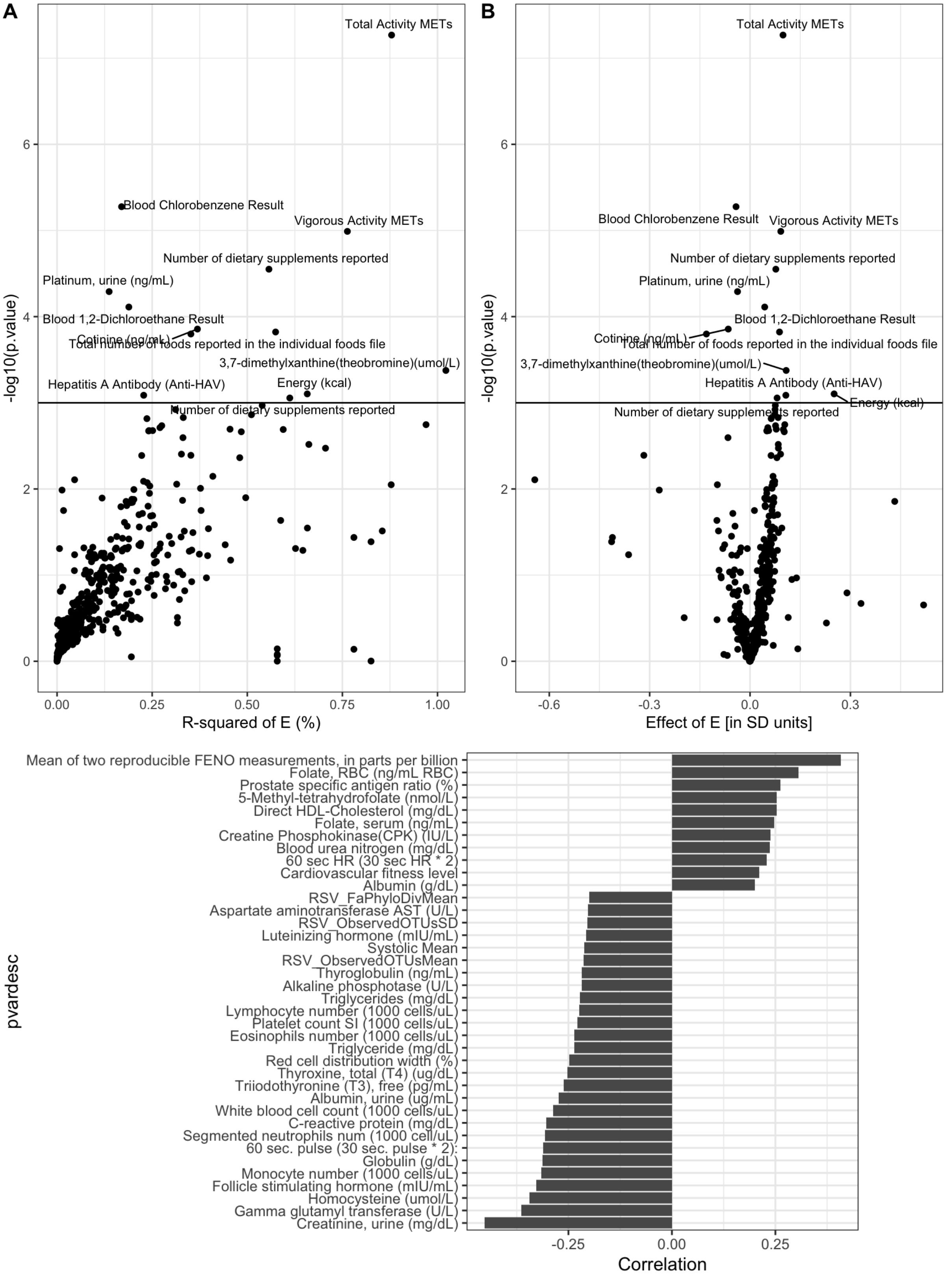

Figure S5. A.) variance explained vs. -log10(pvalue) of exposome variables in association with the digit substitution test after accounting for age, age-squared, race/ethnicity, income, education. B.) Association size (in 1 SD units) vs. -log10(pvalue) for each exposure. C. Shared “exposomic architecture” between the digit substitution test and other phenotypes in NHANES.
